# A Dynamic Ultrasound Phantom with Tissue‐Mimicking Mechanical and Acoustic Properties

**DOI:** 10.1002/advs.202400271

**Published:** 2024-04-22

**Authors:** Sara V. Fernandez, Jin‐Hoon Kim, David Sadat, Colin Marcus, Emma Suh, Rachel Mclntosh, Aastha Shah, Canan Dagdeviren

**Affiliations:** ^1^ Media Lab Massachusetts Institute of Technology Cambridge MA 02139 USA; ^2^ Department of Materials Science and Engineering Massachusetts Institute of Technology Cambridge MA 02139 USA; ^3^ Department of Electrical Engineering and Computer Science Massachusetts Institute of Technology Cambridge MA 02139 USA; ^4^ Department of Mechanical Engineering Massachusetts Institute of Technology Cambridge MA 02139 USA

**Keywords:** dynamic bladder phantom, human‐sized torso tank, mechanical and acoustic properties matching, tissue‐mimicking materials, ultrasound imaging

## Abstract

Tissue‐mimicking phantoms are valuable tools that aid in improving the equipment and training available to medical professionals. However, current phantoms possess limited utility due to their inability to precisely simulate multiple physical properties simultaneously, which is crucial for achieving a system understanding of dynamic human tissues. In this work, novel materials design and fabrication processes to produce various tissue‐mimicking materials (TMMs) for skin, adipose, muscle, and soft tissue at a human scale are developed. Target properties (Young's modulus, density, speed of sound, and acoustic attenuation) are first defined for each TMM based on literature. Each TMM recipe is developed, associated mechanical and acoustic properties are characterized, and the TMMs are confirmed to have comparable mechanical and acoustic properties with the corresponding human tissues. Furthermore, a novel sacrificial core to fabricate a hollow, ellipsoid‐shaped bladder phantom complete with inlet and outlet tubes, which allow liquids to flow through and expand this phantom, is adopted. This dynamic bladder phantom with realistic mechanical and acoustic properties to human tissues in combination with the developed skin, soft tissue, and subcutaneous adipose tissue TMMs, culminates in a human scale torso tank and electro‐mechanical system that can be systematically utilized for characterizing various medical imaging devices.

## Graphical abstract

Various tissue‐mimicking materials (TMMs) for emulating the mechanical and acoustic properties of human bladder muscle, subcutaneous adipose tissue, skin, and soft tissues are developed for medical ultrasound imaging. As a representation, a human‐sized torso tank system with programmable volume control of the dynamic bladder phantom is designed with the purpose to validate ultrasound imaging devices.

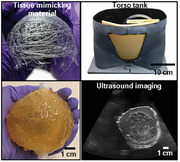

## 1 Introduction

Human tissue phantoms are widely used for characterizing medical devices and advancing academic research. However, many phantoms do not accurately represent the complex, multi‐faceted properties of dynamic human tissues, making them unsuitable models when multiple assessment variables are present.^[^ ^1^, ^2^ ^]^ Ultrasound transducers, for instance, are used by medical professionals to monitor dynamic muscular tissues and organs, such as the human bladder, that possess unique mechanical and acoustic properties relevant for medical imaging.^[^ ^3^ ^]^

The bladder is a hollow, muscular organ that is essential for normal urinary function and overall health. The bladder is composed of three main layers: the urothelium, a thin barrier that prevents urine from entering the bloodstream; the lamina propria, a connective tissue matrix that provides structural stability as the bladder fills; and the detrusor, a relatively thick layer of smooth muscle that contracts to facilitate urination.^[^ ^1^, ^2^, ^4^ ^]^ A 2008 study estimated that over 1.9 billion people were affected by lower urinary tract symptoms, which can be present with a number of disorders including overactive bladder, interstitial cystitis (painful bladder syndrome), bladder outlet obstruction, urinary tract infection, and cancer.^[^ ^5^, ^6^, ^7^ ^]^ Diagnostic imaging plays a crucial role in the diagnosis of such conditions by providing valuable information regarding bladder wall structure, urinary volume, the presence of bladder stones or other obstructions, and the existence of tumors.^[^ ^8^, ^9^, ^10^ ^]^ Because ultrasound is painless, relatively non‐invasive, and does not require radiation exposure, it has been established as a valuable imaging tool for the bladder.^[^ ^10^, ^11^ ^]^

Furthermore, recent ultrasonography techniques and devices show promise in revolutionizing patient comfort in the diagnosis of bladder issues.^[^ ^10^, ^12^ ^]^ For instance, it has been demonstrated that ultrasound shear wave elastography (SWE) can effectively be used to distinguish between compliant and noncompliant bladders, thus providing a non‐invasive and comfortable alternative to traditional urodynamic testing.^[^ ^12^ ^]^ Also, mechanically flexible, even stretchable piezoelectric ultrasound transducers have been developed to overcome the limitations of conventional rigid piezoelectric ultrasound transducers which are not optimally suited for use on highly deformable curved body surfaces due to their rigidity.^[^ ^10^, ^13^, ^14^, ^15^, ^16^, ^17^ ^]^ The design of this conformable patch alternative enables a wearable integration of ultrasound technology with the added benefit of displaying spatio‐temporally precise image reconstruction with a larger field of view for monitoring human tissues at greater depths than conventional ultrasound transducers.^[^ ^13^ ^]^

As new imaging techniques and technologies are explored,^[^ ^10^, ^15^ ^]^ it is critical to ensure that testing and validation can occur in a calibrated and reproducible manner. Imaging phantoms, or objects with known properties that behave similarly to body tissues, provide valuable opportunities to test systems before moving to human subjects, allow for consistent comparison to standard systems, and provide valuable training and teaching opportunities for medical professionals.^[^ ^2^, ^18^ ^]^ For ultrasound and SWE systems, phantoms should properly mimic both the acoustic and mechanical properties of the desired human tissues to ensure realistic imaging results. The stiffness of a material, quantified by its Young's modulus, is the most commonly reported mechanical property used in the development of mechanically accurate phantoms.^[^ ^2^ ^]^ Developing tissue‐mimicking materials with accurate acoustic properties is likewise key to ensuring proper ultrasound imaging results while appropriately representing complex human systems.

Although several experimental and commercially available ultrasound phantoms currently exist for the bladder, none combine the necessary realistic acoustic and mechanical properties with the ability to dynamically change the bladder's volume.^[^ ^2^, ^19^, ^20^ ^]^ CIRS Inc., Norfolk, VA, produces an ultrasound training pelvis phantom composed of their proprietary hydrogel Zerdine and Z‐Skin elastomer. Though these materials have appropriate speeds of sound, acoustic attenuations, and Young's modulus for soft tissues, the volume of the bladder is constant.^[^ ^21^ ^]^ Similarly, in 2010, Ejofodomi, et al. developed a solid gelatin‐based bladder phantom with appropriate mechanical and acoustic properties but a constant volume.^[^ ^2^ ^]^ Wognum, et al. developed a computed tomography (CT) bladder wall phantom with variable volume composed of an excised porcine bladder and water. Although other studies have demonstrated that porcine bladders have similar properties to human bladders, no mechanical or acoustic data was reported for the phantom. Furthermore, as no preservative was used to prevent tissue decay, such a phantom is essentially single use.^[^ ^22^ ^]^ In contrast, a low‐cost, reusable bladder phantom with variable volume was developed by Shellikeri, et al. in 2018 from a latex balloon, silicone, saline, ultrasonography contrast, and psyllium in water. However, no mechanical or acoustic data was reported, and latex does not possess similar properties to the human bladder.^[^ ^1^ ^]^

**Figure 1:**
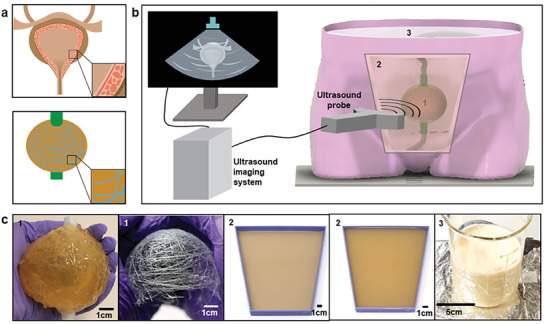
Overview of this work. a) Schematic description shows the structure of the biological bladder (top) and bladder phantom (bottom) fabricated in this work. As shown in the top panel, the real bladder wall is composed of detrusor muscle embedded with collagen fibers. As shown in the bottom image of Figure 1a, the bladder phantom fabricated in this work is composed of muscle TMM embedded with a collagen‐dextran fiber network. As shown here, the bladder phantom fabricated in this work has similar structure with a biological bladder to mimic its dynamic, mechanical, and acoustic properties. b) Schematic description of the torso‐mimicking system for characterizing ultrasound imaging systems. c) Photographs of the various TMMs developed in this work: bladder phantom, collagen‐dextran fiber network, skin TMM, adipose TMM, and soft tissue TMM, respectively (from left).

In this work, we present a design strategy and novel fabrication methods for creating low‐cost and dynamic, tissue‐mimicking materials (TMMs) including bladder phantom as well as other tissues such as skin, adipose (fat), and soft tissues with realistic acoustic and mechanical properties for ultrasound systems. The bladder wall phantom is composed of a collagen‐dextran fiber network attached to a cross‐linked gelatin and psyllium hydrogel, as schematically shown in **Figure** **1**a. A fabrication process was developed to make a hollow ellipsoid‐shaped bladder phantom with inlet and outlet tubes at a low‐cost. Soft tissue is approximated using condensed milk, and a gelatin, agar, and psyllium hydrogel adhered to a urethane rubber panel serves as the subcutaneous adipose and skin TMM. The mechanical and acoustic properties of TMMs were characterized by various methods. To model the overall systems involved with the function of the human bladder inside the body as the bladder expands and contracts upon fluid flow, a life‐sized torso‐shaped container along with an electro‐mechanical system were also developed that mimic the real case as schematically shown in Figure 1b. This bladder wall phantom is suspended in a custom, life‐sized torso‐shaped container filled with a combination of tissue‐mimicking materials representative of soft tissue, subcutaneous adipose tissue, and skin, respectively. A computer‐controlled electronics system enables the user to set the volume of the bladder between 0 and 500 mL.

## 2 Results and Discussion

### 2.1 Physical Property Definitions for Various TMMs

First, we searched the literature for mechanical and acoustic properties of various human tissues to design TMMs with target properties. For increased accuracy, values extracted from literature result from assessments of exclusively human tissues that utilize similar property testing methods to those presented in this work. Mechanical and acoustic properties of subcutaneous adipose tissue, skin, muscle, and soft tissues are listed in **Table** **1**. The human bladder itself has been minimally investigated in the literature,^[^ ^2^ ^]^ leading there to be a wide range of Young's modulus values (70–430 kPa).^[^ ^23^ ^]^ Since the bladder muscle layer in isolation is similar to smooth muscle and has been directly measured in pigs to be ≈16 kPa,^[^ ^2^ ^]^ the target Young's modulus was selected to be at the lower end of the 70–430 kPa human bladder range. Additionally, the Young's modulus of the selected adipose TMM falls above the upper limit of the literature values 3 to 24 kPa to increase fabrication ease and greatly reduce specimen fragility, especially when made with a large form factor.^[^ ^33^, ^34^ ^]^ This further allows the adipose TMM to have more accurate acoustic properties due to the tradeoffs between mechanical and acoustic accuracy as well as account for the effects of adipose tissue in the abdominal cavity.

**Table 1:** List of physical properties of various biological tissues from the literature.

	Young's modulus (kPa)	Speed of sound (m s^−1^)	Attenuation (dB cm^−1^·MHz^−1^)	Density (g cm^−3^)	Impedance (MRayls)
Muscle	70–430^[^ ^23^ ^]^	1547–1616^[^ ^24^, ^25^ ^]^	0.23–1.09^[^ ^24^, ^25^ ^]^	1.04–1.18^[^ ^26^ ^]^	1.65–1.74^[^ ^27^ ^]^
Soft tissue	–	1540^[^ ^28^, ^29^, ^30^ ^]^	0.5–0.75^[^ ^31^ ^]^	1.06^[^ ^26^ ^]^	1.63^[^ ^32^ ^]^
Adipose	3–24^[^ ^33^, ^34^ ^]^	1450–1478^[^ ^24^, ^35^, ^36^ ^]^	0.29–0.48^[^ ^24^, ^35^ ^]^	0.81–0.96^[^ ^26^, ^35^ ^]^	1.35–1.65^[^ ^27^, ^37^ ^]^
Skin	1000–2000^[^ ^38^, ^39^ ^]^	1537–1720^[^ ^36^ ^]^	0.44–1.84^[^ ^40^, ^41^ ^]^	1.10–1.13^[^ ^26^ ^]^	1.53–1.68^[^ ^42^ ^]^

There is wide variation in the literature for skin's Young's modulus which ranges from 0.1 to 18.8 MPa due to different testing methods, hydration levels, skin sites, and tissue heterogeneity.^[^ ^43^, ^44^ ^]^ To uphold a high standard of precision, the selected skin TMM has a Young's modulus which resides within the literature range of 1–2 MPa reported in studies of *ex vivo* human abdominal skin of women aged 43 (± 4) that underwent indentation tests.^[^ ^38^, ^39^, ^44^ ^]^ Note that the model created in the present work is based on CT scan data of a human abdomen of a woman aged 53, and the skin TMM is characterized using indentation tests.

Based on these literature values, we targeted designing TMMs with a Young's modulus of 70 to 430 kPa,^[^ ^23^ ^]^ 3 to 24 kPa,^[^ ^33^, ^34^ ^]^ and 1000 to 2000 kPa^[^ ^38^, ^39^ ^]^ for bladder, subcutaneous adipose, and skin tissue, respectively.

In the same vein, obtaining accurate acoustic property ranges is of particular importance since the phantom system is intended to characterize ultrasound devices while yielding comparable insights to what imaging native tissues would provide. Acoustic properties of the human bladder have been very minimally studied and are largely absent from the literature. Thus, we assumed the acoustic properties of the human bladder to be similar with those of the myometrium, another comparable smooth muscle, due to their similar internal structure.^[^ ^2^, ^36^, ^45^ ^]^ Since soft tissue's speed of sound is assumed to be 1540 m s^−1^ in diagnostic ultrasound imaging^[^ ^28^, ^29^, ^30^ ^]^ and is observed to have attenuations ranging from 0.5 to 0.75 dB cm^−1^·MHz^−1,[^ ^31^ ^]^ we targeted these values in our soft tissue TMM. The representative acoustic properties (speed of sound and attenuation) of various living tissues such as subcutaneous adipose tissue, bladder muscle, skin, and soft tissue are listed in Table 1. Based on these values, we selected proper materials and optimized the fabrication processes to engineer the mechanical and acoustic properties of TMMs, which will be discussed in the next section.

We additionally procured literature values for the densities of each solid tissue of interest to determine what their respective acoustic impedances should be, calculated by multiplying their density and speed of sound values together. Accurately reproducing the acoustic impedance of each tissue is important because this parameter determines the amplitude and phase angle of reflections produced at the boundaries between tissues, as well as the brightness of speckle. Thus, we aimed for a density near 1.04 to 1.18 g cm^−3^, 1.06 g cm^−3^, 0.81 to 0.96 g cm^−3^, and 1.10 to 113 g cm^−3^ for bladder, soft tissue, adipose, and skin TMMs, respectively based on the literature.^[^ ^26^ ^]^ Based on the literature search, we defined the target mechanical and acoustic properties of each TMM. The materials design, fabrication process optimization, and characterization of various TMMs will be discussed in the following section.

### 2.2 TMM Design and Fabrication

To create a complex and biologically realistic phantom that could appropriately assess ultrasound imaging systems, several different TMMs were developed, as shown in Figure 1c. Materials for each TMM were selected based on mechanical and acoustic properties of biological tissues as discussed in the previous section. Gelatin and agar‐based hydrogels were chosen as the primary components of the bladder and adipose TMMs for the following reasons: 1) they closely mimic both the acoustic and mechanical properties of human soft tissue; 2) their properties can easily be tuned by varying weight percentages or through the use of additives; 3) they are relatively easy to manufacture and can be cast into a desired shape with relative ease; and 4) they are inexpensive and easily sourced.^[^ ^46^, ^47^, ^48^ ^]^

For muscle TMM, a gelatin hydrogel was used for the matrix, and formalin was added to increase the Young's modulus by crosslinking the gelatin‐based hydrogels.^[^ ^49^ ^]^ Psyllium was added to increase ultrasound scattering and echogenicity to more closely match its acoustic properties with biological tissues.^[^ ^50^ ^]^ When preparing the muscle TMM, the solution was heated up to 50 °C to fully dissolve the gelatin. For adipose TMM, agar with gelatin hydrogel was used for the matrix, and the ratio of gelatin can be optimized based on the mechanical property of the adipose TMM. Psyllium was also added to the adipose TMM, and diazolidinyl urea was added to prevent bacterial growth. Adipose TMM solution was heated up to 100 °C to fully melt the agar as opposed to the 50 °C used in the gelatin‐only case.

The ratios of different materials — namely deionized (DI) water, gelatin, agar, diazolidinyl urea, and psyllium for the subcutaneous adipose TMM; DI water, gelatin, psyllium, and formalin for bladder muscle TMM; urethane rubber compound (Vytaflex 30, Smooth‐On) Part A and B for the skin TMM; and evaporated milk and diazolidinyl urea for the soft tissue TMM — were optimized based on both literature values and measured mechanical and acoustic properties.^[^ ^50^, ^51^, ^52^ ^]^ The recipe formulations are shown in **Table** **2**. Detailed fabrication procedures for each of the four TMMs and their constituents can be found in the Methods (section 4.1.2.).

**Table 2:** Optimized recipes for TMMs developed in this work. All values in the table are weight ratio.

	DI Water	Gelatin	Agar	Diazolidinyl Urea	Psyllium	Formalin	Evaporated Milk
Muscle	83.7	15.07	–	–	0.59	0.64	–
Soft tissue	–	–	–	0.40	–	–	99.6
Adipose	91.25	6.93	0.91	0.27	0.64	–	–

Although many existing bladder phantoms contain only a single layer of tissue‐mimicking material, the human bladder has a more complex structure, as schematically shown in Figure 1a. To more effectively mimic the mechanical and acoustic properties of the bladder and provide structural stability, a heterogeneously structured, multilayer bladder model composed of muscle TMM, and a collagen‐ and dextran‐based fiber network were conceived of and fabricated. The 3D collagen‐dextran fiber network was fabricated, inspired by tissue engineering fabrics, to be embedded into the muscle TMM.^[^ ^53^, ^54^ ^]^ This work presents the first time in the literature that a collagen‐dextran fiber network has been incorporated into a mechanical or acoustic TMM or phantom system.

**Figure 2:**
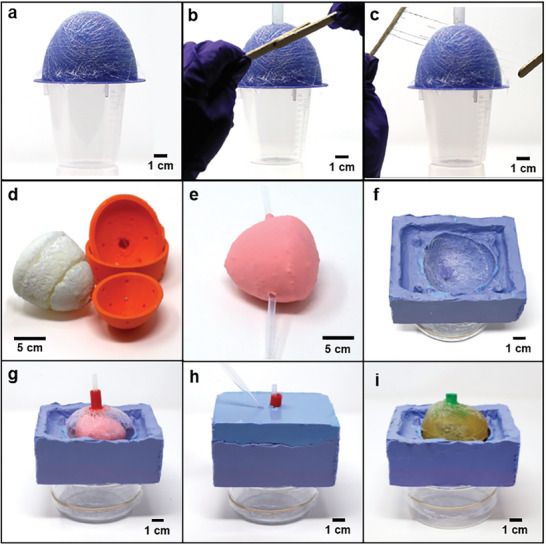
Novel bladder phantom fabrication processes. To fabricate collagen‐dextran fiber network, a) a 3D‐printed mold in the shape of a half bladder was positioned atop a plastic cup. Then, a thin layer of the collagen‐dextran solution was applied to two wood sticks, which were b) pressed together and c) pulled apart slowly to form thin fibers and placed atop the mold in various orientations to form collagen‐dextran fiber network with the shape of a half bladder. d) The water‐dissolvable sacrificial core was prepared, and e) the sacrificial core was glued to two thin plastic tubes and securely wrapped using a balloon. To fabricate the bladder phantom, f) the collagen‐dextran fiber network was placed in the silicone mold (if needed), followed by g. a sacrificial core, another collagen‐dextran fiber network (if needed), and connector tube. h) The top half of the silicone mold was then interlocked with the bottom half, and molten muscle TMM solution was added to the mold using a pipette. After curing the muscle TMM, i) the sacrificial core was dissolved by adding the DI water, and the balloon was removed to obtain a free‐standing hollow bladder phantom.

This collagen‐fiber network fabrication process is described further in the Methods and shown in **Figure** **2**a to c and Figure S1 (Supporting Information). To prepare the collagen‐dextran fiber network, a thin layer of the collagen‐dextran solution was applied to two sterile wooden sticks then pressed together and pulled apart slowly to form thin fibers, as shown in Figure 2b and c. These fibers were draped over the custom 3D‐printed half‐bladder mold to form a cohesive network, as shown in Figure 2c. By using this method, a thin collagen‐dextran fiber network can be deposited layer by layer onto various substrates and the number of layers can be easily controlled. The microstructure of the collagen‐dextran fiber network is shown in Figure S2a (Supporting Information), and one layer of fiber network has an average of 12.4 fibers in 4 mm^2^ with a standard deviation of 2.53. Collagen‐dextran fibers have an average diameter of 8.75 µm, and the corresponding histogram is shown in Figure S2b (Supporting Information).

Although the bladder wall phantom is the primary focus of the imaging system, the tissues surrounding the bladder are similarly important to ensuring realistic behavior for both B‐mode and elastography imaging systems.^[^ ^55^, ^56^, ^57^ ^]^ Selected recipes were thus used to fabricate a full‐size muscle TMM with fibers as well as adipose, skin, and soft tissue TMMs, each with the appropriate form factor as shown in Figure 1c. The process to fabricate the hollow dynamic bladder wall phantom was divided into six steps as schematically described in Figures 2d to i.

To fabricate the full‐size bladder phantom, the sacrificial core was adopted to make a hollow ellipsoid structure. The sacrificial core was composed of sodium bicarbonate (NaHCO_3_), citric acid (HOC(CH₂CO₂H)₂), magnesium sulfate (MgSO_4_), and DI water to make the sacrificial core dissolvable by water. An ellipsoid‐shaped sacrificial core was fabricated by using a 3D‐printed mold (Figure 2d). The overall fabrication procedures of the sacrificial core are shown in Figure S3 (Supporting Information). Plastic tubes were connected to the sacrificial core and a balloon was wrapped around it to both prevent mechanical damage and isolate it from the molten muscle TMM to come (Figure 2e). The core, collagen‐dextran fiber network, and custom‐designed tube connectors were placed into the custom silicone rubber mold (OOMOO 30, Smooth‐On), as shown in Figure 2f and g. The detailed fabrication process for the silicone rubber mold is described in Figure S4 (Supporting Information). Two custom tube connector variations are described in Figure S5 (Supporting Information). Then, the mold was closed and molten muscle TMM was pipetted around this sacrificial core through the fill hole and allowed to cure in an air‐tight wrapping to prepare the mixture for becoming a hollow, ellipsoid‐shaped phantom (Figure 2h). After curing the muscle TMM, DI water was pipetted through the opening at the top of the bladder phantom to dissolve the sacrificial core (Figure 2i). The detailed fabrication step for the bladder phantom is shown in Figure S6 (Supporting Information).

Fabricated bladder phantoms demonstrate mechanical deformability and the ability to be filled with water without leaking, indicating their dynamic properties, as shown in Movie S1 (Supporting Information). As shown in Movies S2 and S3 (Supporting Information), the bladder phantom could be filled with water and emptied, with similar dynamic behavior as biological bladders. These results show high potential for this bladder phantom's utility in various applications.

The soft tissue TMM is fabricated out of evaporated milk (Good & Gather) and diazolidinyl urea (TCI America) with an upscaled formulation of the soft tissue TMM recipe. The skin TMM panel is fabricated using a durable two‐part liquid urethane rubber compound (Vytaflex 30, Smooth‐On) poured into a 3D‐printed trapezoidal mold which is then adhered to the adipose TMM to mimic abdominal skin and subcutaneous adipose tissue, as shown in Figure S7 (Supporting Information). Detailed fabrication procedures for each of the system‐level TMMs can be found in the Methods. Detailed mechanical and acoustic characterization of the muscle TMM used in fabricating the bladder phantom will be addressed in the following section.

### 2.3 TMM Mechanical and Acoustic Property Characterization

**Figure 3:**
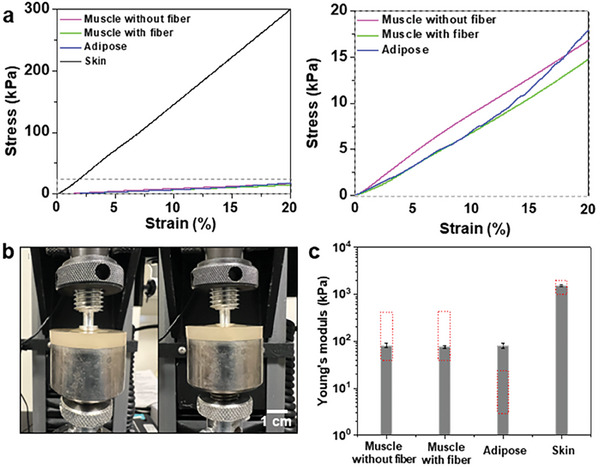
Mechanical property characterization. a) Stress–strain curves of various TMMs measured using compression tests. Left plot shows all TMMs and the right plot shows three TMMs. b) Photograph of the specimen before compression (left) and during compression (right) c) Mechanical properties of various TMMs calculated from Figure 3a. Red dashed boxes indicate the range of reported Young's modulus values from the literature. The exact target Young's modulus values for each TMM are described in Table 1.

Mechanical and acoustic properties were determined experimentally by mechanical compression tests and pulse‐echo experiments. Detailed mechanical and acoustic testing procedures and corresponding data processing steps can be found in the Methods. **Figure** **3**a shows representative stress‐strain curves for TMMs obtained from compression tests. Photographs of the specimen during the compression testing as well as entire testing setups are shown in Figure 3b and Figure S8 (Supporting Information), respectively. To mitigate the effect of ground plane, the specimens were fabricated with a thickness of 6 mm, much thicker than the indentation depth (2 mm). To prevent buckling during the compression test, The ratio of the specimen height to the indented diameter was designed to be less than 1:4.^[^ ^34^ ^]^ Photographs of the specimens are shown in Figure S9 (Supporting Information).

The Young's modulus for each TMM was calculated from the slope of the stress‐strain curve and listed in **Table** **3**. As shown in Figure 3c and Table 3, the skin TMM has a Young's modulus of 1512.7 kPa, which is much higher than the other three tissues. Skin has a higher Young's modulus than other tissue types since in the human body, skin serves as the final barrier to the external environment and must be mechanically robust. As such, the skin TMM's Young's modulus was intended to be higher than other TMMs and match the literature range so that it could serve as a more reliable barrier between the other TMMs and the external environment, while also preventing leakages. Hence, the Young's modulus of skin TMM is 1512.7 kPa, which is similar to the Young's modulus of the skin as shown in Figure 3c.

**Table 3:** Measured physical properties of TMMs developed in this work. Values in parentheses indicate the standard deviation.

	Young's modulus (kPa)	Speed of sound (m s^−1^)	Attenuation (dB cm^−1^·MHz^−1^)	Density (g cm^−3^)	Impedance (MRayls)
Muscle without fiber	81.6 (9.0) [compression] 81.3 (2.2) [tension]	1578.3 (4.8)	0.50 (1.44)	1.04 (0.02)	1.64
Muscle with fiber	75.7 (4.2) [compression] 75.6 (1.7) [tension]	1587.7 (16.0)	5.56 (2.79)	1.06 (0.03)	1.69
Soft tissue	–	1533.4 (0.4)	0.59 (0.01)	1.07 (0.01)	1.65
Adipose	81.2 (10.2)	1507.1 (1.5)	0.48 (0.31)	1.00 (0.02)	1.50
Skin	1512.7 (55.1)	1445.3 (2.8)	2.15 (0.86)	1.03 (0.01)	1.49

Notably, as shown in Figure 3c, the muscle TMM with and without the collagen‐dextran fiber network yielded similar Young's modulus, which indicates that the fibers did not significantly affect the mechanical properties of the matrix. Similar results were obtained from uniaxial tensile testing, as shown in Table 3 and Figure S10c (Supporting Information). The failure strain of muscle TMM without fiber network is ≈80%, however, the muscle TMM with collagen‐dextran fiber network has a lower failure strain of 70%. The reason for a lower failure strain of muscle TMM with fiber network is that the fiber‐muscle TMM matrix interface could behave as a defect for crack propagation.

The reason for the negligible effect of the addition of a collagen‐dextran fiber network on the mechanical property of muscle TMM is that the mechanical testing setup did not sufficiently evaluate the impact of the fiber network on the overall TMM due to geometric constraints. The collagen‐dextran fiber network (on the order of ≈100 µm in thickness) was much thinner than the muscle TMM specimens (≈6 and 2.5 mm thickness for compression and tension tests, respectively) used for mechanical testing. Hence, the overall mechanical properties of the muscle TMM with collagen‐dextran fiber network could be dominated by the primary matrix material, gelatin. To verify the mechanical reinforcement effect of the collagen‐dextran fiber network on the muscle TMM, tensile stress tests with thinner matrix materials can be performed in future.

As shown in Figure 3c, muscle and skin TMM have similar Young's modulus with biological tissues. For the adipose TMM, the Young's modulus of the fabricated TMM is 81.2 kPa, which is somewhat higher than reported values in the literature. This discrepancy comes in avoidance of fabrication and handling issues. In particular, if the Young's modulus of the adipose TMM is optimized by decreasing the weight ratio of gelatin, the fabricated material becomes too fragile and is easily fractured during handling and additional panel assembly steps, especially when a large form factor is used. Hence, a relatively high weight ratio (6.93%) of gelatin is used for adipose TMM to have enough mechanical toughness for handling. One of our future works includes developing adipose TMMs with lower Young's modulus and high fracture toughness.

**Figure 4:**
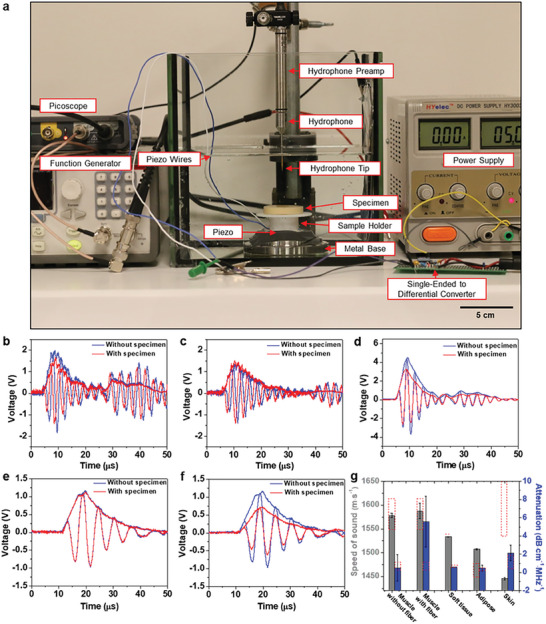
Acoustic property characterization. a) Photograph of acoustic characterization setup. Acoustic measurement results for b) muscle with fiber, c) muscle without fiber, d) soft tissue, e) adipose, and f) skin, respectively. g) Acoustic properties of various TMMs calculated from Figure 4b to f. Red dashed boxes indicate the range of reported values from the literature. The exact target acoustic property values for each TMM are described in Table 1.

Acoustic properties (speed of sound and acoustic attenuation) were measured by using transmission mode pulse‐echo experiments since these are the most relevant and commonly used acoustic properties of soft TMMs for ultrasound imaging.^[^ ^24^, ^58^ ^]^ Other acoustic properties, such as the backscattering coefficient and the nonlinearity parameter, are rarely reported in the literature because they are difficult to measure, and the nonlinearity parameter has a negligible effect on the acoustic properties.^[^ ^24^, ^58^ ^]^ **Figure** **4**a shows an enlarged photograph of the measurement setup. The whole measurement setup is shown in Figure S11 (Supporting Information). As shown in Figure 4a, a single‐ended differential converter was used to remove the electromagnetic coupling and its circuit diagram is shown in Figure S12 (Supporting Information). All measurements were taken in the water tank filled with DI water. The geometry of the specimens for acoustic characterization was the same as those for mechanical characterization, as shown in Figure S9 (Supporting Information). During the pulse‐echo experiment, specimens were inserted into the acoustic path between a piezoelectric transmitter (lead zirconate titanate (PZT)‐based ultrasonic transducer) and a hydrophone receiver, as shown in Figure 4a, and Figure S11 (Supporting Information). Ultrasound waves were generated by the piezoelectric transmitter, and the acoustic signals were recorded by the hydrophone after passing through the specimen. The resonance frequency of piezoelectric transmitter was measured to be 1 MHz using an impedance analyzer, as shown in Figure S13 (Supporting Information). To calculate the acoustic properties of the TMMs, calibration signals were recorded from the pure water tank without the specimen, as shown in Figure S14a (Supporting Information). The detailed acoustic characterization procedure is described in the Methods.

Figure 4b to f shows the voltage readings from the hydrophone during pulse‐echo experiments for various TMMs over time. The blue lines are the calibration waveforms resulting from testing the water tank without a specimen. The red lines are the waveforms resulting from testing the water tank with a specimen of the indicated TMM type. The spatiotemporal offset was corrected to determine the speed of sound in each TMM type, represented here by the perfect horizontal alignment of the blue and red waveforms, shown in Figure 4b to f. Knowing the thickness of specimens and the speed of sound in water (1481 m s^−1^), the speed of sound in the specimens could be calculated using the equation (1):

(1) $$c_{\mathrm{spec}}=\frac{1}{-\frac{\Delta t}{d}+\frac{1}{c_{\mathrm{water}}}}$$

where *c*_spec_, Δ*t*, *d*, and *c*_water_ are speed of sound in the specimen, time offset, specimen thickness, and speed of sound in water, respectively.

The slight difference in amplitude between the peaks of the two waveforms is used to calculate the attenuation. The acoustic attenuation of materials is typically given in units of dB cm^−1^ MHz^−1^. Converting the measured amplitudes to these units was accomplished using the following equation (2):

(2) $$dB_{\mathrm{loss}}=\frac{\left(\frac{20}{100d}\right)\log_{10}\left(\frac{\mathrm{Max}.\mathrm{Pres}\mathrm{sur}e_{\mathrm{cal}}}{\mathrm{Max}.\mathrm{Pres}\mathrm{sur}e_{\mathrm{spec}}}\right)}{f_{c}}$$

where *dB*_loss_, *d*, *Max.Pressure*_cal_, *Max.Pressure*_spec_, and *f*_c_ are acoustic attenuation, specimen thickness, maximum amplitude of the calibration waveform, maximum amplitude of the specimen waveform, and excitation frequency, respectively.

Since a single hydrophone was used to measure the acoustic signal transmitted through the specimen, it is not possible to directly distinguish between signal loss caused by reflection versus attenuation. Therefore, we used the measured density and speed of sound to calculate the acoustic impedance, which was then used to infer the acoustic energy reflection coefficient. The acoustic reflection coefficient is determined by the acoustic impedance of the materials:

(3) $$Z=\rho c_{spec}$$

where Z and ρ are acoustic impedance and density, respectively. The pressure reflection coefficient at the interface of material 1 and 2 is determined as follows:

(4) $$\Gamma =\frac{Z_{2}-Z_{1}}{Z_{2}+Z_{1}}$$

where Γ, Z_1_, and Z_2_ are acoustic pressure reflection coefficient, and acoustic impedance of material 1 and 2, respectively. Hence, the pressure reflection coefficient could be calculated from the speed of sound and density to justify the calculated attenuation values. Note that the sum of acoustic energy reflection and transmission equals one, not the sum of pressure reflection and transmission coefficient. The acoustic energy reflection coefficient is the square of the pressure reflection coefficient, and based on this, the acoustic energy transmission coefficient could be calculated.

The speed of sound of the selected without‐fiber muscle TMM (1578.27 m s^−1^) lies near the center of the 1547^[^ ^24^, ^35^ ^]^ to 1616 m s^−1[^ ^25^ ^]^ range, and the without‐fiber muscle TMM's attenuation (0.25 dB cm^−1^ MHz^−1^) lies at the lower end of the 0.23^[^ ^25^, ^59^ ^]^ to 1.09 dB cm^−1^ MHz^−1[^ ^24^, ^35^ ^]^ range which is preferred since the 0.23 value was measured in vivo. Interestingly, for the muscle TMM with fibers, the speed of sound is similar to that of the muscle TMM without fibers (1587.27 m s^−1^), but the attenuation is notably higher than in the muscle TMM without fiber (5.56 dB cm^−1^ MHz^−1^). Such high attenuation for muscle TMM with fiber networks can be attributed to scattering effects of the heterogeneously arranged fibers acting to disrupt the highly collimated beam from the piezo disk.

Despite this, the structural heterogeneity introduced by the fiber network is expected to cause the muscle TMM to resemble the human bladder more closely during expansion and when acoustically imaging across multiple viewing angles in three dimensions. From these acoustic characterization results, we can provide design rules for muscle TMM. If muscle TMM requires a very accurate attenuation, the fiber network can be excluded from the formulation. If the muscle TMM with similar structural properties is needed, collagen‐dextran fiber networks can be added during the fabrication. This bladder muscle TMM also has the potential to be applied in the formation of other smooth muscle phantoms such as those that resemble the myometrium of the uterus.

The attenuation of the selected adipose TMM lies within the upper part of the 0.29^[^ ^35^ ^]^ to 0.48 dB cm^−1^ MHz^−1[^ ^24^ ^]^ range found in the literature, and its speed of sound is less than 2% greater than the upper end of the 1450^[^ ^35^ ^]^ to 1478 m s^−1[^ ^24^, ^36^ ^]^ literature range, at 1507.1 m s^−1^. This similarity is of particular importance since adipose tissue's comparatively low speed of sound in the human body can introduce image artifacts, especially when located directly below the skin as subcutaneous adipose tissue which is the case during bladder imaging.^[^ ^30^ ^]^ This relationship is well captured by the adipose phantom since its speed of sound at 1507.1 m s^−1^ is notably lower than that of the muscle TMM (1587.7 m s^−1^ for the with fibers case).

For the skin TMM, as mentioned above, in pursuit of more reliable mechanical properties, the acoustic properties are slightly deviated from the measured acoustic properties of human skin as shown in Figure 4g. The selected skin TMM has a speed of sound of 1445.3 m s^−1^, less than 6% below the lower part of the 1537 to 1720 m s^−1[^ ^36^ ^]^ range found in the literature, and its attenuation is less than 1 dB cm^−1^ MHz^−1^ above the 0.44^[^ ^41^ ^]^ to 1.84 dB cm^−1^·MHz^−1[^ ^40^ ^]^ literature range at 2.15 dB cm^−1^ MHz^−1^. Due in part to its homogeneity (no suspended particles), the skin TMM is acoustically distinguishable from the adipose TMM layer it is adhered to which allows for defined barriers between different TMM types during acoustic imaging without affecting the image quality of the muscle TMM itself. Similarly, the selected soft tissue TMM very closely matches the literature targets.

The density of each solid TMM was close to 1 g cm^−3^ which agrees with literature values for bladder muscle, adipose, soft tissue, and skin^[^ ^36^ ^]^ as listed in Tables 1 and 3. Based on the measured speed of sound and density of each TMM, the acoustic impedance was calculated using Equation (1) and summarized in Table 3, and the reflection coefficient was calculated using Equation (2). Based on the calculation, the acoustic energy transmission coefficient at the interface between muscle with fiber, muscle without fiber, soft tissue, skin, and adipose and water is 0.9957, 0.9973, 0.9978, 0.9999, and 0.9999, respectively. Hence, based on this calculation, we can assume that almost 100% of the acoustic loss measured during the pulse‐echo experiments can be attributed to the acoustic attenuation in the TMMs. Thus, using the literature values as a guideline, fabricated TMMs have been confirmed to have similar properties to biological tissues as shown in Figure 3c and Figure 4g where literature value ranges are depicted using dashed red boxes. In combination with these fabricated TMMs, a torso tank system was designed, complete with electronics and corresponding scripts to transform this into a dynamic multi‐TMM system.

### 2.4 Torso Tank System Development

**Figure 5:**
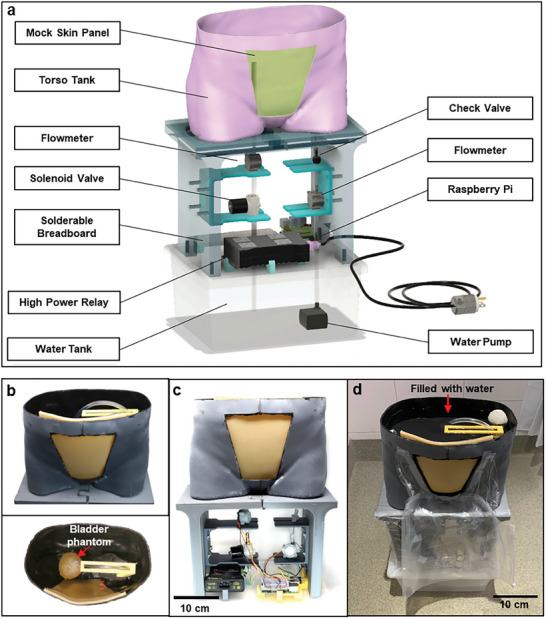
Torso tank system. a) Schematic description of the whole system. b) Torso tank with the skin panel and bladder phantom taken from tilted‐front (left top) and top view (left bottom) c) Front view of the torso tank and middle compartment with electronics. d) Tilted‐front view of the entire system during waterproof test. Torso tank was filled with water and showed no leakage.

Once viable TMMs are fabricated with the proper form factor, they can be used to build a life‐size, dynamic phantom system for assessing ultrasound devices on human tissues to match both human form and function. For the proof of concept, we designed a human‐sized torso tank with peripheral systems for programmable control of the bladder phantom's water content by volume. A schematic description of this system is shown in **Figure** **5**a. A custom torso‐shaped container with a trapezoidal hole in the front was designed using CT scan data of a 53‐year‐old female patient with a BMI of 19.7 that was obtained from The Cancer Imaging Archive's QIN‐Headneck dataset,^[^ ^60^ ^]^ processed, and then 3D printed. Detailed CT data processing procedures are described in more detail in the Supporting Information. This torso‐shaped container is shown in Figure 5b and c.

Shelving and stands were designed, 3D printed, and secured to the lid of the water tank to house electronic components away from potential leaks and guide tubing into both the torso tank above and the water tank below. This middle compartment houses the bladder muscle TMM's electronic fill and release system, complete with a fully functional soldered circuit (schematic circuit diagram could be found in Figure S15, Supporting Information) that accurately controls the flow rate and subsequent volume of water entering or exiting the bladder muscle TMM with sufficient water pressure to reliably maintain a constant flow. A simple console‐based Python script on the Raspberry Pi serves as the user interface for the electronic system, allowing the user to input the desired bladder volume. A schematic description of the electro‐mechanical system of the torso tank is described in Figure S16 (Supporting Information). Further description of the middle compartment construction, electronic components, and Python scripts can be found in the Supporting Information

With the torso tank, electronic components, and water tank in place, these can be combined with the fabricated system‐level TMMs as shown in Figure 5c. The skin and adipose TMM bilayer, which mimics abdominal skin and subcutaneous adipose tissue, respectively, are carefully adhered to each other to make the skin panel, as shown in Figures S7 and S17 (Supporting Information). This combined panel is then carefully attached to the inside of the torso tank by adding glue (3 M Scotch‐Weld Plastic & Rubber Instant Adhesive, PR1500) to the outward‐facing perimeter of the skin TMM and allowed to cure for several days (as shown in Figure 5b and Figure S17 (Supporting Information)). To prevent liquid leakage, liquid rubber neopond sealant was further applied to the seam to fill any gaps. A waterproofing test was performed as described in Supporting Information and shown in Figure 5d and Figure S17c,d (Supporting Information). Then, the bladder phantom is subsequently placed into the torso tank (as shown in Figure 5b), followed by the addition of the soft tissue TMM. The complete fabricated phantom system is shown in Figure S17 (Supporting Information).

**Figure 6:**
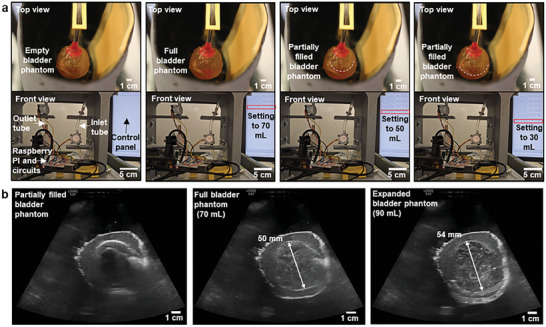
Operation of the torso task system with a bladder phantom. a) Photographs of the bladder phantom connected to the torso tank system with an empty (the first column), full (the second column), and partially filled (third and fourth columns) bladder phantom, respectively. The bottom photos show the electromechanical systems and a control panel for controlling the volume of the bladder phantom. b) Ultrasound images of the bladder phantom while partially filled (the first image), full (the second image), and expanded (the third image), respectively.

As shown in **Figure** **6a** and Video S4 (Supporting Information), an electromechanical system could programmable control the volume of the water in the bladder phantom multiple times with high reliability. The desired water volume in the bladder can be entered into the Raspberry Pi using a keyboard and monitor. Then it will determine whether it needs to put additional water into or drain water from the bladder to achieve the desired volume. If the Raspberry Pi is to add more water to the bladder, it operates the pump to pull the water from the reservoir. When the Raspberry Pi is to remove the water from the bladder, a solenoid valve opens to drain the water from the bladder. During operation, two flow meters connected to the inlet and outlet tubes, respectively, measure the volume of the water to determine when to stop the pump or close the valve. As shown in Figure 6a, the bladder phantom was fully filled by adding 70 mL of water, and also could be partially filled with a desired volume.

The torso tank system was also used for ultrasound imaging of the bladder phantom using a commercial ultrasound imaging system (GE LogiqE10), as shown in Figure S18 (Supporting Information). For the ultrasound imaging of the bladder phantom, the collagen‐dextran fiber network was not incorporated due to its adverse effect on the acoustic properties, as mentioned above. As shown in Figure 6b and Video S5 (Supporting Information), when the bladder is not full, the full bladder wall could not be observed in the ultrasound image due to large reflection by air trapped within the phantom. When the bladder phantom was filled with water using the electromechanical system in the torso tank, all bladder walls of the phantom could be clearly identified. It was also confirmed that the bladder phantom can expand its volume ≈28.6% (from 70 to 90 mL) by the addition of water. Upon adding an extra 10 mL of water, the connector‐bladder phantom interface broke, as observed in Figure S19 (Supporting Information). It should be noted that the low adhesion between bladder phantom and a 3D‐printed connector (shown in Figure S5, Supporting Information) is the weakest point. One of the key future works would be improving the adhesion by adopting a surface treatment or hydrogel‐based wet adhesive.^[^ ^61^, ^62^ ^]^

Table S1 (Supporting Information) provides a comparison of the bladder phantoms reported in the literature. As shown in Table S1 (Supporting Information), the bladder phantom developed in this work has the closest mechanical and acoustic properties to the human bladder. Furthermore, owing to the torso tank system, our bladder phantom can be programmably filled and emptied, and expanded. The torso tank demonstration verified that this system can be used to characterize various ultrasound devices including conventional ultrasound probes and novel conformable ultrasound patches alike.^[^ ^10^ ^]^ This can also be used as a training tool for aspiring ultrasound technicians to gain confidence before assessing human tissue directly.

## 3 Conclusion

In this paper, we present various novel TMMs and their fabrication processes that culminate in a dynamic bladder phantom system with accurate mechanical and acoustic properties and behaviors. We set target physical properties for the tissues of interest and designed TMMs based on corresponding Young's modulus, density, speed of sound, and attenuation values in the literature. Namely, this work developed bladder muscle, soft tissue, adipose, and skin TMMs with realistic mechanical and acoustic properties as well as 3D collagen‐dextran fiber networks that could be used to fabricate system‐level phantoms with realistic form factors for characterizing ultrasound transducers. The TMMs were characterized via mechanical and acoustic tests, and the data was processed using unique scripts to extract relevant property values.

The bladder phantom combined the bladder muscle TMM with and without collagen‐dextran fiber networks and utilized a first‐of‐its kind dissolvable sacrificial core to yield a hollow interior suitable for filling with, storing, and expelling liquid during elastic deformation of the phantom. The muscle TMM recipe can be applied to create a phantom of any smooth muscle, such as the myometrium of the uterus, provided that the muscle's mechanical and acoustic properties are sufficiently similar to those of the bladder. The collagen‐dextran fiber network can be optionally included in accordance with desired properties, behaviors, and applications. The adipose and skin TMMs were also formed into a dual‐layer panel of the appropriate thickness, referred to as the skin panel for brevity. These system‐level phantoms were combined with a CT scan‐based torso tank and a fully functional electronics system that programmatically controls the flow rate and subsequent volume of water entering or exiting the bladder phantom.

In the future, there are a few additions to be made to the system. From a materials design standpoint, further experimentation is needed to produce a skin TMM with more realistic acoustic properties without compromising the system's mechanically robust, contamination‐preventing barrier to the external environment. The system's soft tissue TMM could similarly be made more complex to account for specific soft tissues that reside within the torso, rather than serving as an approximation for abdominal soft tissue. Likewise, further experimentation is needed to develop an adipose TMM with lower Young's modulus and high fracture toughness.

With this multi‐TMM bladder phantom system, ultrasound transducers of different form factors can be characterized outside of clinical settings, leading to more streamlined protocols for assessing medical device performance. This system additionally provides a means for clinicians‐in‐training to practice operating ultrasound transducers on a realistic torso phantom under different dynamic bladder‐fill conditions with a mechanically and acoustically accurate user experience for the first time. We anticipate that this work will inspire other researchers to create mechanically‐ and acoustically‐realistic tissue phantoms of different form factors and will instruct them on how to fabricate dynamic, hollow hydrogel forms in pursuit of more anatomically‐accurate, functional representations of biological systems.

## 4 Experimental Section

### TMM fabrication

*Collagen‐dextran fiber network fabrication*: 3D collagen‐dextran fiber networks were fabricated to emulate tissue engineering fabrics, with some modification,^[^ ^53^, ^54^ ^]^ in preparation for being embedded into the muscle TMM. DI water was pipetted into 1 mL of Type 1 Bovine Telocollagen solution (10 mg mL^−1^, Advanced BioMatrix) until the combined mass reached 1.24 g. The collagen‐water solution was then mixed by hand until becoming a homogenous solution. Following this, 1.24 g of dextran (molecular weight 500 000, Spectrum Chemical) was also manually stirred into the solution until the mixture was uniform, and the top of the container was sealed to prevent the mixture from drying out. This mixture of Telocollagen, DI water, and dextran was then left on a shaker in its mixing container at 1200 rpm speed for 24 h. This time could be reasonably reduced as needed to speed up fabrication.

To create the network, a thin layer of the collagen‐dextran solution was applied to two sterile wooden tongue depressor sticks, which were then repeatedly pressed together and pulled apart slowly to form thin fibers. These fibers were then draped taught over the outside of a custom 3D‐printed half‐bladder mold. A small piece of tubing was optionally added to the top of the mold to result in a small hole at the top of the fiber network. For optical microscopy analysis, collagen dextran fibers were deposited on the Si wafer. This process was repeated by varying orientations and positions to create a dense, multi‐directional fiber network in a desired 3D shape with a consistent thickness of at least 100 µm. The collagen‐dextran fiber network was allowed to dry on the mold for 24 h before removal. This fabrication process is shown in Figure 2 and Figure S1 (Supporting Information).

### TMM Fabrication Procedures

*Adipose*: Mock subcutaneous adipose tissue was fabricated from 6.93% powdered gelatin (Aldon Corporation), 0.91% powdered agar (Aldon Corporation), 0.64% psyllium (Metamucil) to introduce scattering, 0.27% diazolidinyl urea (TCI America) to prevent bacterial growth, and the remaining 91.25% DI water by mass. For a small batch, this corresponds to 3.8 g powdered gelatin, 0.5 g powdered agar, 0.35 g psyllium, 0.15 g diazolindyl urea, and 50 mL of DI water, and this recipe could be upscaled as needed.

First, all powders were weighed, placed into a clean glass bowl (VWR) wiped with IPA, and stirred with wooden tongue depressor sticks to break up clumps. A hot plate was set to 100 °C. Then, the desired volume of DI water was measured in a plastic graduated beaker (VWR) and added to the powders before being stirred by hand with the wooden tongue depressor stick for ≈30 s to prevent initial clumping. The mixture was then covered with cling film (Saran Wrap) and microwaved in 10‐s increments, ≈40 s for the small batch, until the mixture achieved a low viscosity and had reached ≈90 °C. This time varied depending on the volume of TMM being fabricated in a given batch. Then, the plastic covering was removed and stirred lightly with a wooden tongue depressor stick, breaking up large clumps. A magnetic stirrer was added, and the bowl was covered tightly with aluminum foil. Pulling the foil taut, the temperature probe was used to create a small hole in the foil. This hole was then used to periodically check the temperature of the mixture during the stirring process and adjust the hot plate accordingly. When not actively probing the mixture, the hole was covered with a small piece of aluminum foil.

The glass bowl was then transferred to a magnetic hot plate set to 90 °C and stirred at 100 rpm for 1 min, 200 rpm for 9 min, 300 rpm for 25 min, and 400 rpm for 8 min. The molten mixture was then removed from the hot plate and allowed to cool to 60 °C, about two min, while sitting covered for 1 min to allow any steam to condense back into the mixing bowl.

Finally, the molten mixture was poured into custom cylindrical acrylic molds lightly greased with canola oil (PAM) and allowed to cure at room temperature in a plastic bag (VWR) for 16 h before removal to preserve the moisture content and prevent dehydration prior to testing, ensuring that the surface of the bag did not contact the top of the samples during the curing process. These custom cylindrical acrylic molds were used to create samples for mechanical and acoustic testing, and their construction is described in Supporting Information and shown assembled in Figure S9 (Supporting Information). For the system‐level adipose TMM, this recipe was upscaled, and a larger mixing container was used.

### Bladder Muscle

Mock bladder muscle tissue was fabricated from 15.07% powdered gelatin (Aldon Corporation), 0.59% psyllium (Metamucil) to introduce scattering, 0.64% formalin (37% Formaldehyde, Ward's Science) to prevent bacterial growth, and the remaining 83.7% DI water by mass. For a small batch, this corresponds to 9 g powdered gelatin, 0.35 g psyllium, 0.38 g formalin, and 50 mL of DI water, and this recipe could be upscaled as needed.

A well‐mixed, molten solution of gelatin, psyllium, and DI water was created using the same procedure described above for mock subcutaneous adipose tissue but with a few notable changes. The temperature of the hot plate was set to 50 °C rather than 100 °C and adjusted during the stirring process to maintain a mixture temperature of 50 °C. Also, toward the end of the stirring of the molten mixture on the hotplate, formaldehyde solution was pipetted into a clean plastic beaker until the desired mass was achieved. After the molten mixture was removed from the hot plate, the formaldehyde solution was added and stirred by hand with a wooden stir stick for ≈1 min prior to adding to the custom acrylic molds.

Cylindrical specimens with and without the collagen‐dextran network were fabricated to compare the effect of the fiber networks on the mechanical and acoustic properties. Dogbone‐shaped specimens were prepared for the tensile tests. In the case where fibers were included, a flat collagen‐dextran fiber network was placed flush with the bottom of the mold before the molten mixture was poured. For the system‐level muscle TMM, this recipe was upscaled, and a larger mixing container was used.

### Soft Tissue

Mock liquid soft tissue was fabricated from 99.6% evaporated milk (Good & Gather) and 0.40% diazolidinyl urea (TCI America) to prevent bacterial growth by mass. For a small batch, this corresponds to 350 mL of evaporated milk and 1.4 g of diazolidinyl urea, and this recipe could be upscaled as needed.

Liquid generic mock soft tissue was fabricated using evaporated milk (Good & Gather) and diazolidinyl urea (TCI America) to prevent bacterial growth. The desired volume of evaporated milk was measured in a plastic graduated beaker (VWR) and poured into a clean glass bowl (VWR). Then, the desired amount of diazolidinyl urea was weighed and added to the evaporated milk. The mixture was then stirred at 500 rpm with a magnetic stirrer at room temperature for 15 min. This was stored in a labeled airtight container at room temperature. For the system‐level soft tissue TMM, this recipe was upscaled, and a larger mixing container was used.

### Skin

Mock skin was fabricated using a durable two‐part liquid urethane rubber compound (Vytaflex 30, Smooth‐On). Per manufacturer's instructions, equal parts A and B by volume were dispensed into plastic cups and mixed individually by hand with a wooden stir stick for ≈30 s. Then, both parts were added to a separate plastic beaker and mixed with a wooden stir stick for 3 min. To create cylindrical test samples to measure mechanical and acoustic properties, the mixture was poured into custom acrylic molds greased with a thin layer of petroleum jelly (Vaseline). Alternatively, cylindrical silicone molds (BAKER DEPOT) could be used to circumvent the risk of urethane bonding to plastics during the curing stage. The compound was allowed to cure at room temperature for at least 16 h before being demolded. Note that although this recipe is commonly known and urethane‐based materials have been used extensively to simulate mechanical properties of human skin,^[^ ^63^, ^64^ ^]^ this recipe has yet to be applied in isolation to create a skin TMM to mimic the acoustic properties of human skin.

### Skin and Adipose Panel

The skin TMM panel is fabricated using a durable two‐part liquid urethane rubber compound (Vytaflex 30, Smooth‐On) poured into a 3D‐printed trapezoidal mold up to the first fill line, granting it a thickness of 2 mm. The adipose TMM panel was made by pouring the corresponding mixture over the cured skin TMM up to the second fill line, granting it a thickness of 1.581 cm. Both TMMs were gently unmolded following curing, and the skin TMM was placed on a clean, even surface. To adhere the two TMMs, one person uses a wooden tongue depressor stick to apply a thin layer of glue (3 M Scotch‐Weld Plastic and Rubber Instant Adhesive, PR1500) to the perimeter of the skin TMM's top surface little‐by‐little in preparation for the addition of the cured adipose TMM. Concurrently, the adipose TMM is held gently with two hands supporting it from beneath, and slowly laid on top of the skin TMM in a rolling motion, taking care to maintain contact between the adipose and skin TMMs once they touched and ensuring that no air was trapped between the two layers. This panel fabrication is shown in Figure S7 (Supporting Information).

### Bladder Phantom Mold Design

The process to fabricate the bladder wall phantom was divided into six steps: 1) Fabricating a custom PLA model of the phantom bladder, 2) Creating a custom silicone mold, 3) Fabricating a sacrificial core, 4) Positioning the sacrificial core within the mold, 5) Casting molten mock muscle into the mold with tubing and fibers within, and 6) Dissolving the sacrificial core for unmolding. First, two hollow tri‐axial half ellipsoids with wall thickness 3 mm were designed in AutoCAD since the thickness of unexpanded human bladder walls has been measured to be between 2.2 and 4.4 mm for men and 2.5 to 4.4 mm for women.^[^ ^65^ ^]^ The two halves of the ellipsoid were given different heights to mimic the bladder's flattened shape when empty. The top and bottom bladder halves were given dimensions a = 3.25 cm, b = 2.75 cm, c = 2 cm and a = 3.25 cm, b = 2.75 cm, c = 4.75 cm, respectively, to achieve an unexpanded volume of ≈125 mL. A circular hole was added at the top and bottom of the ellipsoid to allow the attachment of tubing. The ellipsoid was 3D‐printed in PLA plastic. Arbitrarily crumpled aluminum foil was uncrumpled and wrapped around the ellipsoid model and secured with tape. This adds texture reminiscent of rugae, the anatomical folds found on the surface of the human bladder. The PLA ellipsoid was then used to create a custom two‐part mold from a pourable silicone rubber compound (OOMOO 30, Smooth‐On) as shown in Figure S4 (Supporting Information) and described in more detail in Supporting Information.

### Sacrificial Core Fabrication

A sacrificial, shape‐customizable core material must be used to create a hollow, continuous (no seams) bladder. A water‐soluble sodium bicarbonate core material was selected to provide the necessary structural and mechanical integrity of the inner mold during curing and handling while allowing for quick and easy dissolution by a continuous stream of room temperature DI water during de‐molding. A dry mixture was created with 4.5 parts (by volume) baking soda (NaHCO_3_) (Arm and Hammer), 3 parts citric acid (HOC(CH₂CO₂H)₂) (Milliards) and 1 part Epsom salt (MgSO_4_) (up & up) and stirred thoroughly for 5 min in a glass beaker to create a homogenous mixture and break up clumps. 1.5–2 mL of DI water (per tsp of Epsom salt in the mixture) were added drop‐by‐drop through a pipette while stirring continuously. With nitrile gloves donned, the mixture was combined thoroughly using fingers to crush and knead it for 5 min.

The inner surface of the plastic molds was sprayed with Pam's cooking spray which was spread evenly around the mold surface with a Kim wipe. A small wooden stick was placed through the two opposite holes in the mold. A 1 tsp measure was used to transfer the mixture into the mold. After every 1–2 spoonfuls, the mixture was pressed down lightly with fingers to pack it gently against the sides of the mold without leaving any air pockets, avoiding packing the mixture too tightly. Once filled, the molds were transferred into a vacuum oven and dried at 65 °C for 6 h. The prepared cores were removed by holding the mold open‐face down and tapping firmly on a flat table‐top. The three parts of the inner core were tacked together using a few spots of Scotch‐Weld (3 M PR40, clear) and allowed to sit at room temperature for 30 min. This process is shown in Figure S3 (Supporting Information).

### Sacrificial Core Assembly

The bulbs of two 7.5 mL transfer pipettes (VWR‐16001‐188) were cut off, and the remaining tube‐like portions were inserted into the sacrificial core, one on either side, and pushed gently until firmly secured. A few spots of Scotch‐Weld (3 M PR40, clear) were added if needed, making sure to avoid blocking the opening of the pipettes. A small slit (≈2 mm) was added at the top of a rubber balloon in preparation for wrapping the balloon around the core. The mouth of the balloon was stretched as wide as possible, and the mouth of one of the modified pipettes was guided in through the incision. The balloon was gently but firmly pulled taut over the entire body of the sacrificial core. Another tiny incision was made directly next to the mouth of the balloon which was then carefully stretched so that the topmost tube no longer went through the mouth of the balloon but rather though the tiny incision at the base of the balloon's mouth. This minimizes excess balloon material at the base of the thin tube and makes secured pieces lay flatter. The excess balloon material was then cut off and the remaining material around the pipette was secured using glue (Scotch‐Weld, (3 M PR40, clear)) such that the sacrificial core was no longer exposed. The prepared core was suspended from a benchtop clamp, and the entire surface of the balloon was sprayed with resin spray (Castin’ Craft Resin Spray, TAP Plastics) allowing for appropriate ventilation. This was allowed to dry for 30 min. This process is shown in Figure S3 (Supporting Information).

### Bladder Mold Preparation

The bladder mold preparation process begins prior to fabricating the bladder muscle TMM. For repeatability and ease in later steps, this process should be performed in a fume hood. First, the top opening of a clean glass bowl (VWR) wiped with IPA was sealed using a tautly pulled sheet of cling film (Seran wrap) secured with a rubber band. The vessel must have a height great enough to enclose the length of the tube connector (≈2 cm), and a diameter (10–12 cm) appropriate for stably supporting the weight and size of the finished mold assembly. A small incision (2‐3 mm) is made in the center of the cling film (Seran wrap) in preparation for the bottom tube connector, which is plugged with sulfur‐free clay. Next, the inner surface of both halves of the bladder mold are lightly greased with canola oil (PAM), and the bottom half is placed centered atop the glass bowl (VWR). One collagen‐dextran fiber network is then placed inside the bottom half of the mold, followed by a 3D‐printed TPU tube connector, the sacrificial core assembly, a second collagen‐dextran fiber network, and a second tube connector, ensuring that the tube connectors pass through the holes in the collagen‐dextran fiber networks and the bladder mold halves, respectively, and are positioned to suspend the sacrificial core at the proper height. A generous quantity of petroleum jelly (Vaseline) is then added onto the mold's seams using a wooden tongue depressor stick to prevent leaking during the fill process. The two bladder mold halves are then interlocked, and any gaps are coated with additional petroleum jelly (Vaseline) as needed. Note that this bladder mold should be prepared partially within a large, open bag such that it can be separated from the air during the muscle TMM cure process without disturbing the mold itself. This fabrication process is shown in Figure S6 (Supporting Information). The tube connectors are shown in Figure S5 (Supporting Information). Note that the tube connector in Figure S5a (Supporting Information) is better suited for orthogonal phantoms, whereas the tube connector in Figure S5b (Supporting Information) with a more curvilinear design is used in the final version of the bladder phantom.

### Bladder Phantom Fabrication

The bladder muscle TMM was prepared following the fabrication steps delineated above. Then, a pipette was used to transfer the mixture from the glass bowl (VWR) to the fill hole at the top of the mold, ensuring that no bubbles were transferred. The mixture was placed on the hot plate as needed to prevent premature curing. This slower, more controlled method of pipetting as opposed to pouring minimizes trapped air and allows for more immediate intervention if leaks begin to occur. If there were no leaks, the mold was continually filled until full, taking care to not overfill the pour spout. Then, the mold assembly was enclosed in a large plastic bag or cling film (Seran wrap) using tape such that it was isolated from the air and allowed to cure for 16 h. If mild leaks occur, these could often be resolved by doing one or a combination of the following: 1) proceeding as normal, waiting for the leaked material to begin to cure as it may sufficiently plug holes, 2) adding additional petroleum jelly (Vaseline) to the seams, 3) adding an additional layer of the mold‐making material (OOMOO 30, Smooth‐On) in problematic areas (at the seams) and allowing to cure.

Once the muscle TMM was cured, the procedure to dissolve the sacrificial core could start. Leaving the two halves of the mold assembly intact, the clay plug was gently removed from the bottom tubing connector, taking care not to tug on the exposed tube connector. Once the fluid path through the pipettes appears clear (no clogged clay or glue), the sacrificial core is gradually dissolved by introducing a continuous stream of DI water through the mold using another large pipette or a 30 mL syringe. DI water was added slowly so that the fizz produced by the dissolving sacrificial core does not exit through the top of the mold. The dissolved solution should flow out of the tubing at the bottom of the mold and accumulate in the glass container below. Water is continuously streamed through the core until the solution coming out of the bottom runs clear. If the core becomes overly dehydrated, DI water can be mixed with the materials used to make the core and streamed through it. Once the core is fully dissolved, a pair of tweezers could be used to gently pull on the top and bottom pipettes to remove them along with the encapsulating balloon through the tubing connectors, taking care to not pierce the bladder phantom. Then, the two halves of the mold are gently separated, pushing down slightly on the tube connector to prevent tearing it off. The bladder phantom complete with attached tube connectors are carefully removed from the mold, the clay is cleaned out of the tube connectors, and the bladder phantom is stored in a sealed plastic bag.

### TMM Characterization Methods

*Mechanical property characterization*: The material stiffness of the specimens was determined experimentally by mechanical compression testing on an Instron 5943 Universal Testing System (Instron, Inc., Canton, MA) using a load cell rated to 10 Newtons (Figure S8, Supporting Information). Cylindrical and dogbone specimens were prepared by using acrylic molds. The geometry of the cylindrical specimen is 40 mm diameter by ≈6 mm thickness, as shown in Figure S9 (Supporting Information). The dogbone specimen has a total length of 140 mm and a thickness of 2.5 mm, and the elongated region has a 59 mm length and 6 mm width, as shown in Figure S10a (Supporting Information). The fabrication procedure for the acrylic mold was described in Supporting Information. All specimens were tested within 5 days of fabrication (i.e., after curing).

During the compression test, the displacement and load into the top surface of the specimen were recorded, while the bottom compression platen remained stationary. After the specimen was loaded onto the Instron tester, vegetable glycerin was applied to the surface of the specimen as a lubricant to prevent the specimen from sticking onto the indenter during testing. This was used to prevent sudden slips or load measurement spikes while under compression due to buckling. The indenter was lowered to make initial contact with the specimen's top surface to zero the instrument displacement and load. The elastic modulus of the viscoelastic specimen is not constant and is dependent on the precompression applied. This study does not use a precompression. The Instron test was programmed for a total compressive displacement of 2 mm at a rate of 0.5 mm min^−1^. Load and displacement data were recorded at 40 Hz. Following testing, any residue was properly removed from the Instron.

The ratio of the specimen height to the smallest dimension in the plane perpendicular to the height was no more than 1:4.^[^ ^34^ ^]^ This geometry prevented specimens from buckling during compression when deformations up to 30% were applied. Thus, a diameter of 10 and 40 mm were chosen for the indenter and specimen, respectively.

For the tensile test, the specimens were loaded into the Instron device using screw‐side action grips with a rubber coating on the grip pad. Specimens were stretched until they failed with a stretching rate of 500 mm min^−1^. The Instron load and displacement data were converted to engineering strain and engineering stress and plotted. The slope of the stress versus strain graph was taken between 10%−20% strain to ensure a linear region of curve fit and so that recordings began after the initial stabilizing effects of the indentation test.

### Acoustic Property Characterization

The material speed of sound and attenuation at 1 MHz were determined experimentally by acoustic testing using an Onda needle hydrophone HNC‐0400 with inline preamplifier AG‐2010 connected to an oscilloscope (PicoScope 5444B). A 1 MHz piezoelectric element of PZT was acquired, and a thin circular sheet of Ecoflex (Smooth‐On) was cured around it for ease of handling. The resonant frequency of the piezoelectric element was confirmed to be 1 MHz using an impedance analyzer (Keysight E4990A) as shown in Figure S13 (Supporting Information).

The Ecoflex‐coated piezoelectric element was fixed to a circular steel plate using electrical tape to prevent it from floating, and then the assembly was placed at the center of a glass water tank. A tubular plastic stand was similarly taped to the metal plate, creating a platform where specimens could be placed above the piezoelectric element without obstructing the acoustic beam path. The plexiglass box of dimensions 20 cm by 20 cm by 20 cm was then filled with DI water to a depth of ≈10 cm, and a fixture was used to hold the needle hydrophone vertically such that its tip was submerged enough to prevent interference from acoustic reflections from the water surface (≈1.5 cm). The hydrophone was connected to the oscilloscope, and the PicoScope 6 software was set to use AC coupling with a voltage range of ± 100 mV, sampling rate of 125 MSPS, and the 20 MHz bandwidth limit enabled. A math channel was then configured to progressively average the received signal to achieve an improved signal‐to‐noise ratio (SNR). Finally, the oscilloscope was set up to trigger using the sync signal from the function generator to eliminate any timing jitter.

The piezoelectric element was driven as follows. A Keithley 3390 function generator was set to generate single‐cycle sine bursts at a 1 MHz center frequency and 100 Hz repetition rate. This single‐ended output was then converted into a balanced 8 Vpp differential signal by a custom circuit using the MCP6292 dual operational amplifier. The piezoelectric element was driven by the differential signal, which helped reduce electromagnetic interference (EMI) coupling through the water to the hydrophone. To further reduce EMI, a GND‐coupled sheet of aluminum foil was immersed in the water tank.

After waiting for surface ripples to subside, the oscilloscope trigger was enabled and allowed to progressively average the received signal until a low‐noise signal was acquired (typically 5–10 s). The acoustic signal typically reached the hydrophone with a 30–40 µs delay, indicating an acoustic path length of 4.4–5.9 cm, consistent with the dimensions of the plexiglass box.

The testing procedure involved first acquiring a calibration waveform with only water in the acoustic path, in order to observe the propagation delay and received signal amplitude with a material of known properties (1481 m s^−1^ and 0 dB loss). Next, a specimen was inserted into the acoustic beam path using tweezers and gloved hands while being careful not to disturb the testing setup. The new propagation delay and amplitude were then recorded. This data acquisition process was repeated for all solid TMM specimens, with additional calibration waveforms taken as needed.

Liquid soft tissue TMM was tested using a similar setup and procedure. The piezoelectric element and hydrophone were positioned in the same way, with the same circuits and equipment. The calibration waveform was collected as before, with pure water in the acoustic path. The water was then removed from the container by siphoning (to avoid disturbing the setup) and replaced with the liquid soft tissue TMM, and the specimen waveform was acquired. The same equations were used to determine speed of sound and attenuation, with the only difference that the “specimen thickness” (d) was set to equal the entire distance between the piezoelectric element and hydrophone tip.

To measure the density of the soft tissue TMM, soft tissue TMM was fabricated as described above. Then, the mass of 50 mL of soft tissue TMM was measured using a scale to calculate the density. This measurement was performed three times to check the consistency of values.

The acoustic waveforms were recorded using the PicoScope 6 software and saved as .csv files for later analysis by a custom Python script. In the script, paired specimen and calibration waveforms are first loaded as 1D Numpy arrays. These waveforms are expected to have similar shapes, with time delays and differences in amplitude corresponding to differences in the speed of sound and attenuation, respectively.

Before analysis, the data needed some preparation. First, the signal had a residual DC component despite the oscilloscope being nominally AC‐coupled. This DC component would cause problems in later steps, so it was removed by subtracting the mean. Second, the sampling rate of the oscilloscope was 125 MSPS, corresponding to an 8 ns time resolution. Experimentally, we often needed to measure time delays < 100 ns, and thus the native time resolution would be too coarse. To mitigate this issue, the waveforms were upsampled by a factor of 8 to give 1 ns resolution, using the “resample_poly” function from SciPy. This method was chosen to avoid any edge effects that might be caused by FFT‐based resampling.

The difference in propagation time between the specimen and calibration waveforms was measured by taking the cross‐correlation and finding the index where the maximum value occurred. This index was then converted into the time difference by multiplying it with the sampling period of 1 ns. Finally, Equation (1) was used to calculate speed of sound in the specimen.

The attenuation caused by the specimen was determined by measuring the difference in waveform amplitude between the calibration and specimen data. Measuring the amplitude was accomplished by the following method. First, each waveform was multiplied with a Hann window in the time domain, with the window centered at the peak of the acoustic pulse. The Hann window was chosen because it provides a nearly‐flat central region for an accurate amplitude measurement, while tapering to zero at the edges to suppress FFT edge effects. After windowing the signal, a Hilbert transform was used to obtain the complex‐valued extension of the waveform, followed by taking the absolute value to get the envelope. The maximum value of the envelope was then compared between the specimen and calibration waveforms to determine the attenuation. For similar impedance (Z) values, the reflection coefficient was confirmed to be extremely small, indicating that reflected energy had a negligible impact on the attenuation measurements. The acoustic attenuation of materials is typically given in units of dB cm^−1^ MHz^−1^. Converting the measured amplitudes to these units was accomplished using the Equation (2).

## Conflict of Interest

The authors declare no conflict of interest.

## Author Contributions

S.F. and J.‐H.K. contributed equally to this work. C.D. conceived the research idea, designed the research direction and directed all research activities. S.F., R.M., and C.D. designed the research methodology and aims. S.F. performed an extensive literature search to tabulate accurate mechanical and acoustic properties of human tissues. C.M. provided theoretical and practical ultrasound insights that informed design criteria. S.F. and E.S. fabricated cylindrical TMM samples and fiber networks. D.S. and J.‐H.K. created acrylic TMM molds and performed mechanical characterization. S.F. and C.M. performed acoustic characterization testing and wrote corresponding data processing scripts. S.F. processed the acoustic data. A.S., S.F., and J.‐H.K. fabricated sacrificial cores. S.F. and J.‐H.K. fabricated TMM molds and system‐level TMMs. R.M. designed the system circuit and corresponding scripts, and S.F. soldered electronic components in accordance with the design. S.F. and J.‐H.K. assembled the system. D.S. and J.‐H. K. prepared photograph‐containing figures, J.‐H.K. prepared quantitative figures, and E.S. prepared 3D models and fabrication process illustrations. S.F. and J.‐H.K. finalized the project in alignment with author guidelines. All authors contributed to manuscript writing.

## Supporting information

Supporting Information

Supplemental Movie 1

Supplemental Movie 2

Supplemental Movie 3

Supplemental Movie 4

Supplemental Movie 5

## Data Availability

The data that support the findings of this study are available from the corresponding author upon reasonable request.
