# Supplementary material for: A Dynamic Ultrasound Phantom with Tissue‐Mimicking Mechanical and Acoustic Properties
Source: Adv Sci (Weinh). 2024 Apr 22;11(22):2400271. doi: 10.1002/advs.202400271 (PMC11165531; doi:10.1002/advs.202400271)
Supplement: Supplementary file 1 — Supporting Information [file ADVS-11-2400271-s006.pdf]

ADVANCED SCIENCE
Open Access

Supporting Information

for *Adv. Sci.*, DOI 10.1002/advs.202400271

A Dynamic Ultrasound Phantom with Tissue-Mimicking Mechanical and Acoustic Properties

*Sara V. Fernandez, Jin-Hoon Kim, David Sadat, Colin Marcus, Emma Suh, Rachel McIntosh, Aastha Shah and Canan Dagdeviren**

Supporting Information

A Dynamic Ultrasound Phantom with Tissue-Mimicking Mechanical and Acoustic Properties

*Sara V. Fernandez, Jin-Hoon Kim, David Sadat, Colin Marcus, Emma Suh, Rachel Mclntosh, Aastha Shah, and Canan Dagdeviren**

Table of Contents

Supplementary Notes
S1. Bladder mold fabrication.
S2. Acrylic mold fabrication for compression test specimen preparation.
S3. Torso tank design and validation.

Supplementary Figures S1 to S19

Supplementary Table S1

Supplementary Information References

Movies S1 to S5

Supplementary Notes

S1. Bladder mold fabrication.

First, an open-top mold box with at least 1 inch of clearance around the edges of the ellipsoid was created using five laser-cut plexiglass sheets that were hot glued together, ensuring that all seams were completely sealed (Figure S4a). Parts A and B of the silicone rubber compound were mixed with a volume ratio of 1:1 by hand with a wooden stir stick to make a homogeneous mixture (Figure S4b and c). An ellipsoid mold was prepared by 3D printer (Figure S4d). The ellipsoid model was then wrapped with arbitrary-crumpled aluminum foil (Figure S4e) and placed into the center of the mold box, wide-side down, and pushed into the clay (Figure S4g). Then, the bottom layer of the mold box was filled with an even layer of sulfur-free clay that was approximately 1.27 cm thick (Figure S4f). Additional clay was used to secure the ellipsoid model at its base and to bury its bottom half.

An additional approximately 1.27 cm thick and 1.27 cm tall clay wall was added to the inner perimeter of the box (Figure S4g). This creates a positive extrusion that will interlock and mate with its negative counterpart contained within the second half of the mold, thereby creating an additional higher surface-area seal around the hollow chamber to prevent leakage during the addition of molten bladder muscle TMM during the bladder phantom fabrication process.

A pour spout was created by attaching a small cylinder of clay on the plastic half ellipsoid close to the tubing hole (Figure S4g). A similar clay cylinder with a diameter smaller than the intended tubing size was added as a continuation of the tubing hole (Figure S4g). Thinning out the clay cylinders by applying pressure gently outward from the base greatly improves adhesion to the aluminum foil. Registration keys were added to each corner of the mold box by creating a small, dome-shaped hole in the clay with the end of a wooden stir stick. The silicone was then poured slowly into the mold box to completely cover the ellipsoid, taking care to avoid completely submerging the pour spout or clay tube extension, and was allowed to cure for at least six hours until fully cured (Figure S4h).

To create the second half of the mold, the mold box was carefully disassembled using a utility knife for glue removal and forceful panel separation. All clay was then removed from the box and ellipsoid, and these were cleaned with IPA. The first half of the silicone mold was then flipped over with the ellipsoid placed flush in the mold, and the mold box was reassembled around the mold using hot glue as before (Figure S4i). A thin layer of petroleum jelly (Vaseline)

was applied to the surface of the silicone mold half prior to pouring to ensure that the two halves would not bond together during the curing process. A clay cylinder with a diameter smaller than the intended tubing size was again added as a continuation of the tubing hole (Figure S4i). Once again, parts A and B of the silicone rubber compound were mixed with a volume ratio of 1:1 by hand with a wooden stir stick until the color of the mixture was uniform before being poured into the mold box until the ellipsoid was completely submerged with at least 1.7 cm of clearance, taking care to avoid completely submerging the clay tube extension (Figure S4j). The second mold half was allowed to cure for at least 6 h until fully cured. The mold box was then disassembled, the plastic ellipsoid was removed, and the mold was cleaned with IPA (Figure S4k and l).

S2. Acrylic mold fabrication for compression test specimen preparation.

During fabrication, the specimens were cast in custom acrylic molds and cured at room temperature. This mold ensured that the bottom surface of the specimens were as flat as possible, making fabrication more repeatable and consistent. The multi-part mold further allowed for seamless unmolding as screws could be removed, and the bottom layer could be twisted off with ease without damaging the specimens. An image of the custom acrylic mold can be found in Figure S9. The mold was fabricated by laser cutting 1.7 cm acrylic sheets. A circular bottom layer acrylic sheet served as the flat bottom of the mold, while a slightly smaller middle layer was laser cut to 40 mm inner diameter to define the specimen shape. Concentric screw holes were added into all layers to enable the mold assembly to be clamped and sealed prior to pouring the liquid solution.

S3. Torso tank design and validation.

3.1. Preparation of 3D torso tank model using CT scan data.

CT scan data of a healthy 53-year-old female patient with a BMI of 19.7 was obtained from The Cancer Imaging Archive’s QIN-Headneck dataset[1]. This data was converted from DICOM format to NRRD format using 3D Slicer to preserve patient anonymity[2]. NRRD data was then converted to STL format with Democratiz3D (Embodi3D) using the “Skin” operation[3]. The resulting STL file was cut to the torso region and hollowed to 3 cm thickness using Autodesk Meshmixer[4].

3.2. Conversion of CT scans to STL files.

Firstly, the NBIA Data Retriever was installed to convert files into DCM format. The appropriate data was downloaded from the Cancer Imaging Archive's QIN-HEADNECK Dataset. The Data Access tab was accessed by scrolling down and clicking the "Search" button to open the Data Portal. In the Simple Search filters, the following were selected: QIN-Headneck (Collections), CT (Image Modality), and ABDOMEN (Anatomical Site). The Search Results tab was used to select data, and that data was added to the cart by clicking the cart icon in the same row as the intended subject ID. Data was downloaded from the cart by clicking the Download button. In this way, the data was automatically download with the NBIA Data Retriever. The downloaded files were opened, a directory was selected in which to place them, and the start button was clicked to download them to the selected directory.

The 3DSlicer App was installed to convert the CT scans into NRRD format. Data was converted by following the general instructions by embodi3D. Different cross sections of the uploaded CT scans could be displayed by toggling the slide bars in each box.

Democratiz3D on embodi3D were used to convert the NRRD files to STL files. A free account was created to access the application. Democratiz3D was launched, and the appropriate NRRD file was uploaded to be processed. File information was entered and appropriate options were selected within the Type and Privacy settings. "Skin" was selected under Operation and the appropriate processing parameters and processed file options were chosen. Terms of Use were accepted and Submit was clicked. File processing completed and then the processed file could be accessed. Once the STL file had been generated, an email with a link to the file was sent to the email associated with the account. The file was accessed via the website as follows. The user profile was clicked and "My Files" was selected to see a list of relevant files. Both the source files that the user uploaded and the processed files were shown here. The STL was downloaded by selecting the posted file and clicking the "Download this file" button.

Autodesk Meshmixer was installed to edit the mesh STL. The proper STL file was then imported into the app. To crop the mesh model, "Edit" and then "Plane Cut" were selected. The plane was adjusted to preserve the correct section of the mesh and a Fill Type was selected to make the cut surface open or closed. To make the entire model solid or hollow, "Edit" was selected followed by either "Make Solid" or "Hollow." To extrude a surface of the mesh, "Select" was selected and

then the intended surfaces of the model were highlighted by clicking and dragging over them. Then, “Edit…” and “Extrude” were selected. The settings were adjusted as needed. Following the conclusion of editing, the model was exported as an STL. Lastly, the STL file was opened in the CAD software. This method is compatible with any CAD software. This could be repeated for male models or models with different BMIs.

3.3. 3D Torso tank fabrication.

The top of the container was left open for easy access to the inside of the torso model and to allow upward volumetric displacement of the soft tissue TMM that would fill the torso model. A trapezoidal hole was designed as part of the ventral side of the container in preparation for the mock skin panel to be adhered to it using glue.

The maximum build volume of the Prusa i3 MK3 used to print each of the PLA plastic components of the torso tank is 25 cm by 21 cm by 21 cm. Due to these dimensional constraints of the 3D printer, the container was printed in two halves, with the cutting plane defined as extending from the dorsal side to the ventral side of the torso. The two separate halves were then bonded together with liquid rubber neopond sealant (Liquid Rubber Canada), and any holes or topographical irregularities were sanded with a variable speed rotary tool (Dremel, Mt. Prospect, Illinois, U.S) to reopen and smoothen them, respectively.

Holes were drilled into the base of the torso tank to accommodate tubing to be attached to the bladder TMM. One hole was near the center of the torso for easy routing to the bottom of the bladder TMM and the other was placed to the side to correctly route the tube that was to pump water into the top of the bladder TMM.

Following this, the inner surface of the torso-shaped container was coated with 13 layers of the liquid rubber neopond sealant brushed on in thin coats and allowed to cure for at least 6 hours between coats, or until the previous layer was tacky. The addition of several layers of liquid rubber neopond sealant greatly minimizes reverberations in the tank that can lead to acoustic artifacts. A 3D-printed tube-guiding stand was also glued to the inside of the tank. The torso tank was then adhered to the top of a custom 3D-printed shelf that had been printed in two parts and adhered using the liquid rubber neopond sealant with holes in alignment with those on the torso tank. Note that when cleaning the surface, isopropyl alcohol (IPA) should be avoided since contact with alcohol causes the rubber coating to wrap and peel.

3.4. Electro-mechanical system development.

Three stands were designed to house electrical components including a relay, a Raspberry Pi computer and Pi Hat, and a high power relay. Each stand was made to fit the footprint and contours of each component and 3D printed with PLA material. The stands could be glued in fixed positions on the lid of a water container to allow for easy organization and portability of the electronics, and their elevated nature reduces the risk of water exposure to the electronic components. The lid of the water container has a hole cut out of it to allow for the insertion of a funnel for easy filling at later stages. Modular shelves were also bolted on walls opposite from each other in the middle compartment to house the solenoid valve, check valve, and flowmeters in the torso tank system. The shelves guide the tubing between the torso tank and the water container and properly space the valves and flowmeters above the electronics. Each tubing hole was given five thou tolerance when designed and was filed down if a greater tolerance was needed using a variable speed rotary tool (Dremel, Mt. Prospect, Illinois, U.S.). The shelves were designed to allow the valves and flowmeters to slide in and out of notches for easy access and maintenance. These components are shown in Figure 5c.

A Raspberry Pi 3 Model B was used to control the electronic bladder fill and release system with the circuit soldered onto an attached PiHat. Schematic circuit diagram is shown in Figure S13. A submersible pump (WP550-3, GROWNEER) in a container of water beneath the torso could be turned on via a high-power relay (DLI 705020645490, Digital Loggers, Inc.) to fill the bladder phantom with water. A one-way check valve (Blulu) was used to prevent water backflow from the bladder to the submersible pump. A solenoid valve (RSC-1-12VDC, ESV) controlled via relay (SRD-DC03V-SL-C, HiLetgo) was placed under the bladder phantom to control water flow out of the bladder. Excessive voltage spikes from turning the solenoid on and off were prevented via flyback diode. Hall-effect sensor flowmeters (Seeed Technology Co., Ltd) were used to determine the water flow rate in and out of the bladder in $mL \cdot s^{-1}$. This flow rate was determined by counting the number of pulses received from the flowmeter in one second and multiplying by a conversion factor provided by the manufacturer. The volume of water entering or exiting the bladder was calculated by multiplying the water flow rate by the number of seconds that had passed since beginning to fill or empty the bladder, respectively. A voltage divider was used to normalize the flowmeter waveform to safe voltage levels for the Raspberry Pi. A simple console-

based Python script on the Raspberry Pi served as the user interface for the electronic system, allowing the user to input the desired bladder volume.

3.5. Waterproofing test for the whole torso tank system.

Following visual inspection and minor touch-ups to the skin panel's adhesion to the torso tank, the system is righted and tested for water impermeability while ensuring that electronics are protected from any unforeseen water exposure using plastic bags and cling film. This is vital to ensure that the skin and adipose TMM panel is successfully separating the essential components of the bladder phantom from the external environment and protecting them from disturbance and contamination. This test demonstrating that the system is completely leak-proof is shown in Figure 5d and Figure S17c and d.

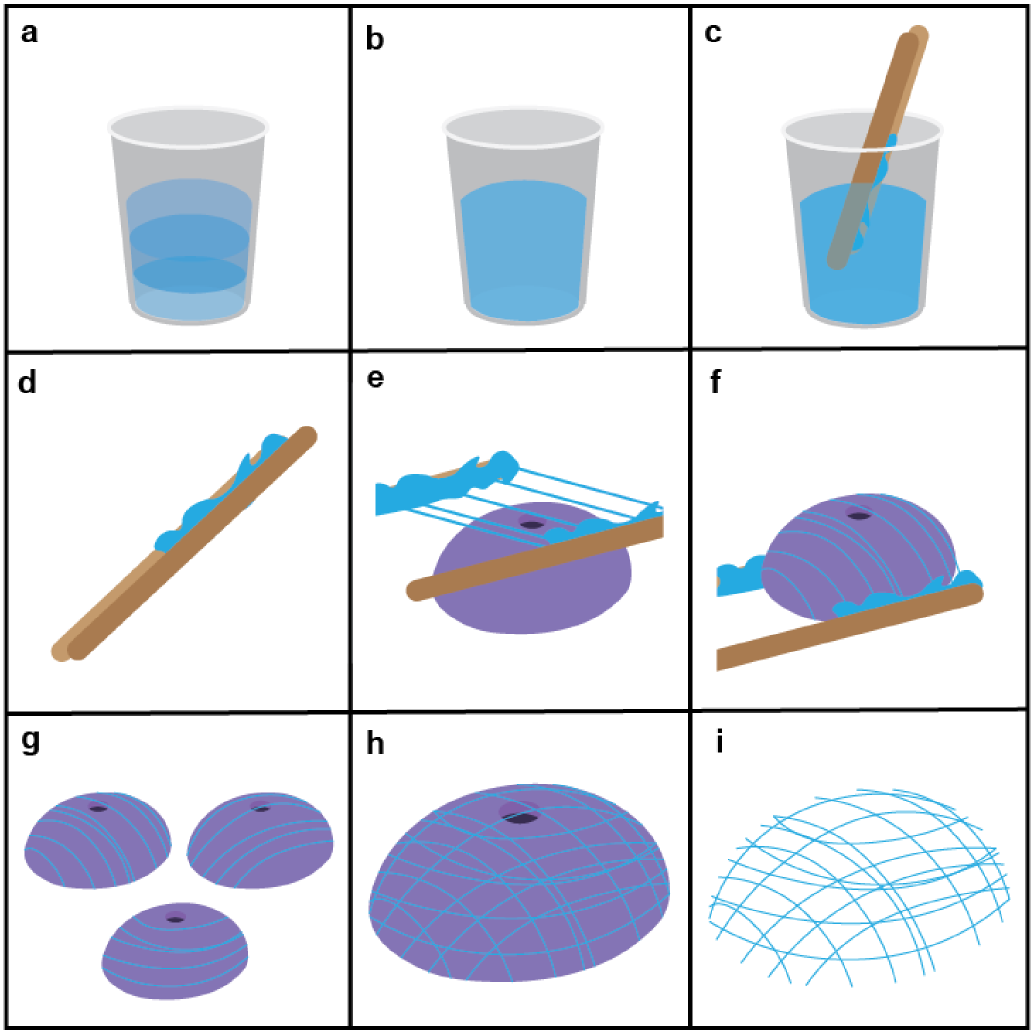

Supplementary Figure S1. Schematic description of 3D collagen-dextran fiber network fabrication. a. Collagen, water, and dextran were combined in the proper ratios and **b.** left on a shaker overnight to make a homogeneous solution. **c.** A thin layer of the collagen-dextran solution was applied to two sterile tongue depressor sticks, **d.** which were then pressed together, **e.** pulled apart slowly to form thin fibers and placed atop the mold in various overlapping orientations (**g.** aligned and **h.** random networks) to form **i.** the 3D collagen-dextran fiber network in the shape of the bladder half.

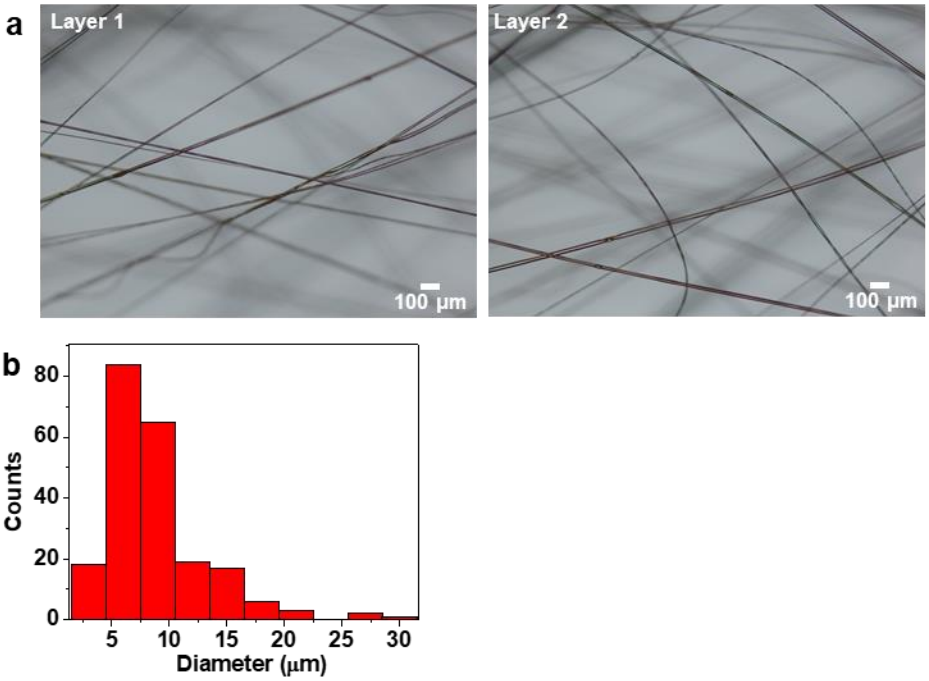

Supplementary Figure S2. Microstructural analysis of 3D collagen-dextran fiber network. **a.** Optical microscopy images of different layers of the collagen-dextran fiber networks. **b.** Diameter distribution of the collagen-dextran fibers from 215 fibers.

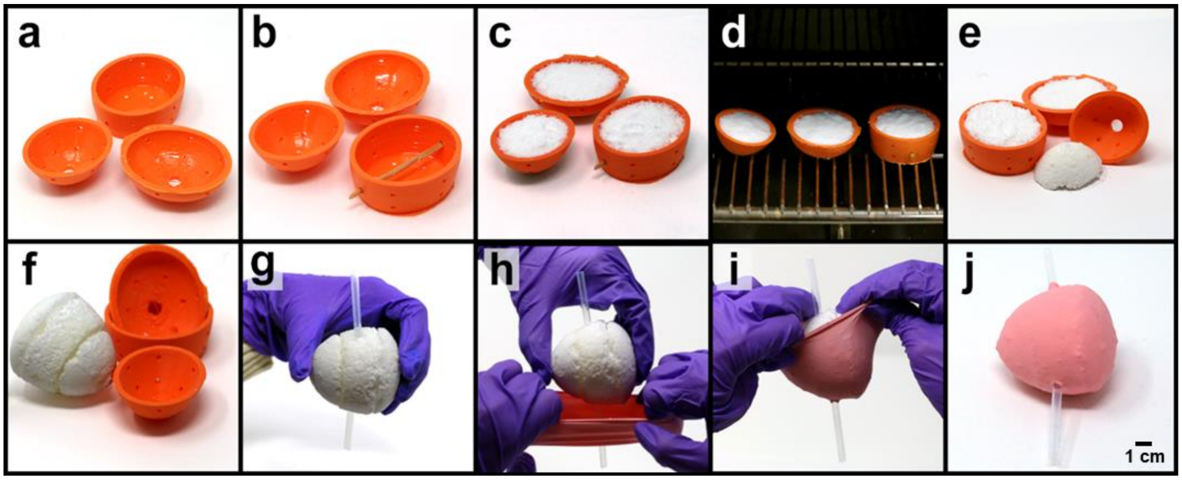

Supplementary Figure S3. Sacrificial core fabrication. a. A three-part PLA mold with **b.** a small wooden stick spanning across two opposite holes was **c.** filled with the water soluble mixture and **d.** placed in a vacuum oven to dry at 65°C for 6 hours. **e.** The three sacrificial core segments were unmolded and **f.** glued together. Following curing, **g.** tube-like portions of small transfer pipettes were added to the sides of the sacrificial core in preparation for **h to j.** securing a balloon around the sacrificial core.

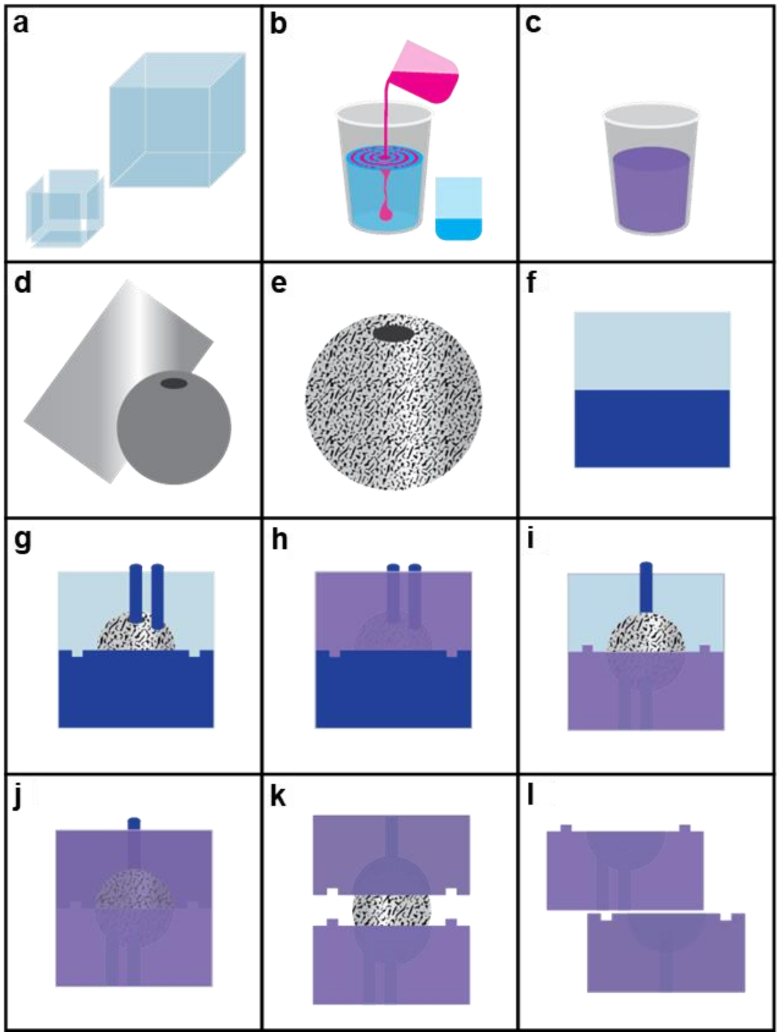

Supplementary Figure S4. **Schematic of bladder mold fabrication. a.** Five laser-cut plexiglass sheets were hot glued together to create an open-top box. **b.** Two parts of a pourable silicone rubber compound (OOMOO 30, Smooth-On) were combined and **c.** mixed to make a homogeneous solution. **d.** Prepare ellipsoid bladder model using 3D printer and aluminum foil. **e.** Aluminum foil was arbitrarily crumpled and wrapped around the 3D-printed ellipsoid bladder model. **f.** Clay was added to the bottom of the plexiglass box. **g.** Registration keys and an overflow channel were added, and the aluminum-foil-wrapped ellipsoid was added along with two clay cylinders, one as a continuation of the tubing hole, and the other to form the pour opening beside it. h. The silicone mixture was poured into the box, allowed to cure, and removed from the box by deconstructing it. **i.** The mold was flipped upside down and added into the reconstructed box, and the ellipsoid and clay cylinder were added. Petroleum jelly (Vaseline) was added to the surface of the cured mixture. **j.** A second batch of the mixture was then added

until the ellipsoid was submerged but before the clay was completely covered. **k.** The assembled clay was allowed to cure, **l.** and then the ellipsoid and clay were removed.

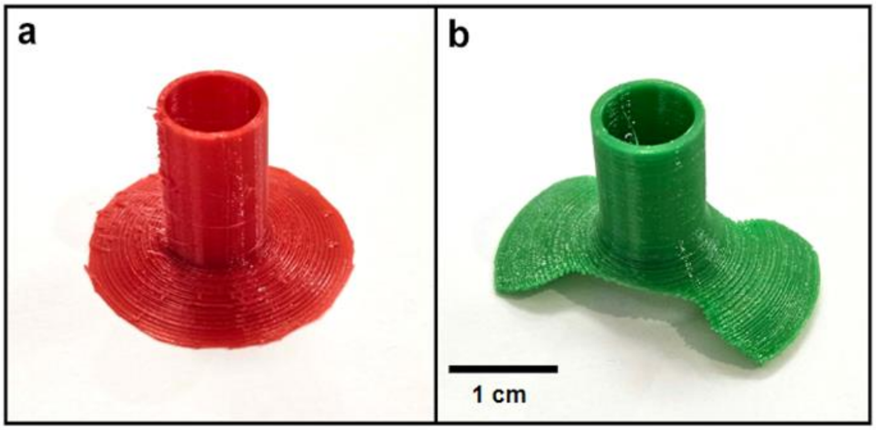

Supplementary Figure S5. Custom tube connectors design. a. The more planar tube connector is suitable for use with hollow orthogonal phantoms, whereas **b.** the curvilinear tube connector is designed to conform to the contours of the sacrificial core and is therefore more compatible for integration with the bladder phantom. Thus, this second, more ergonomic design was utilized for the final version of the bladder phantom.

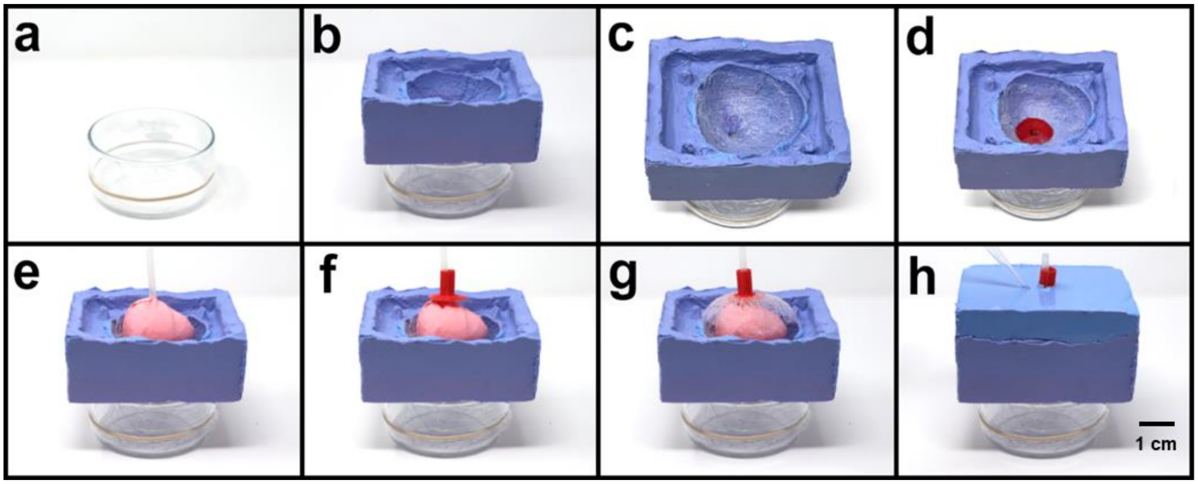

Supplementary Figure S6. System-level bladder TMM fabrication. a Prepare a glass container with a layer of tautly-pulled cling film secured by a rubber band. **b.** A prepared silicone mold (fabrication steps are schematically shown in Figure S3) is placed atop the cling film on the glass container. **c.** A collagen-dextran fiber network is placed into the silicone mold. **d.** A tubing connector plugged with clay is placed through the hole that passes through the collagen-dextran fiber network (Figure S5c), silicone mold (Figure S5b), and cling film (Figure S5a**). e.** The sacrificial core is placed atop the bottom tubing connector, followed by **f.** the top tubing connector, **g.** the complementary collagen-dextran fiber network, and **h.** the second half of the silicone mold, complete with petroleum jelly (Vaseline) applied to the seams. The bladder TMM mixture is subsequently added to the mold through the top fill hole using a pipette, as shown in Figure S5h.

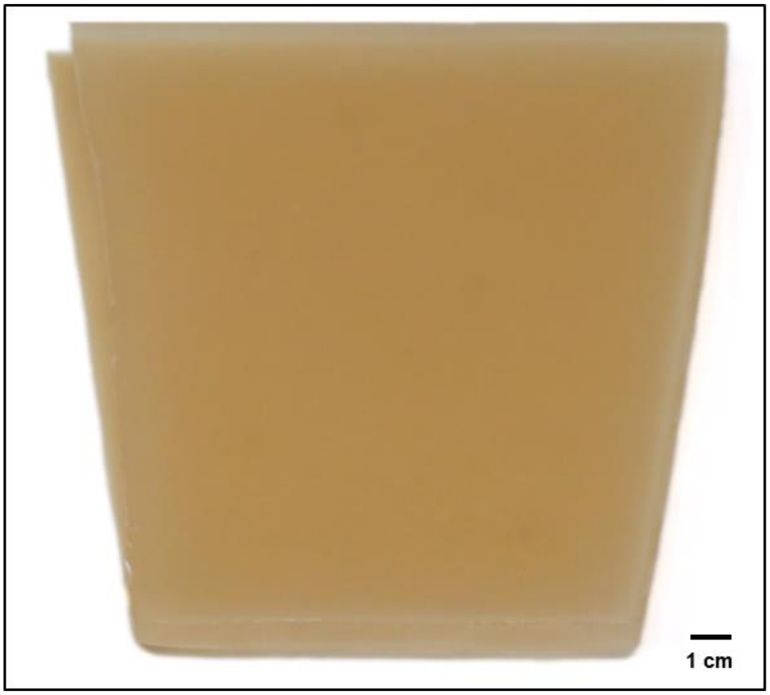

Supplementary Figure S7. Skin panel integration. A prepared skin TMM and adipose TMM are attached to make an abdominal tissue. To attach skin and adipose TMM, a thin layer of glue around the seams of the skin TMM panel, the adipose TMM is placed on top of it in a rolling motion beginning at the top to prevent trapping air between the two surfaces. For simplicity, this entire structure is referred to as the “skin panel” in the text.

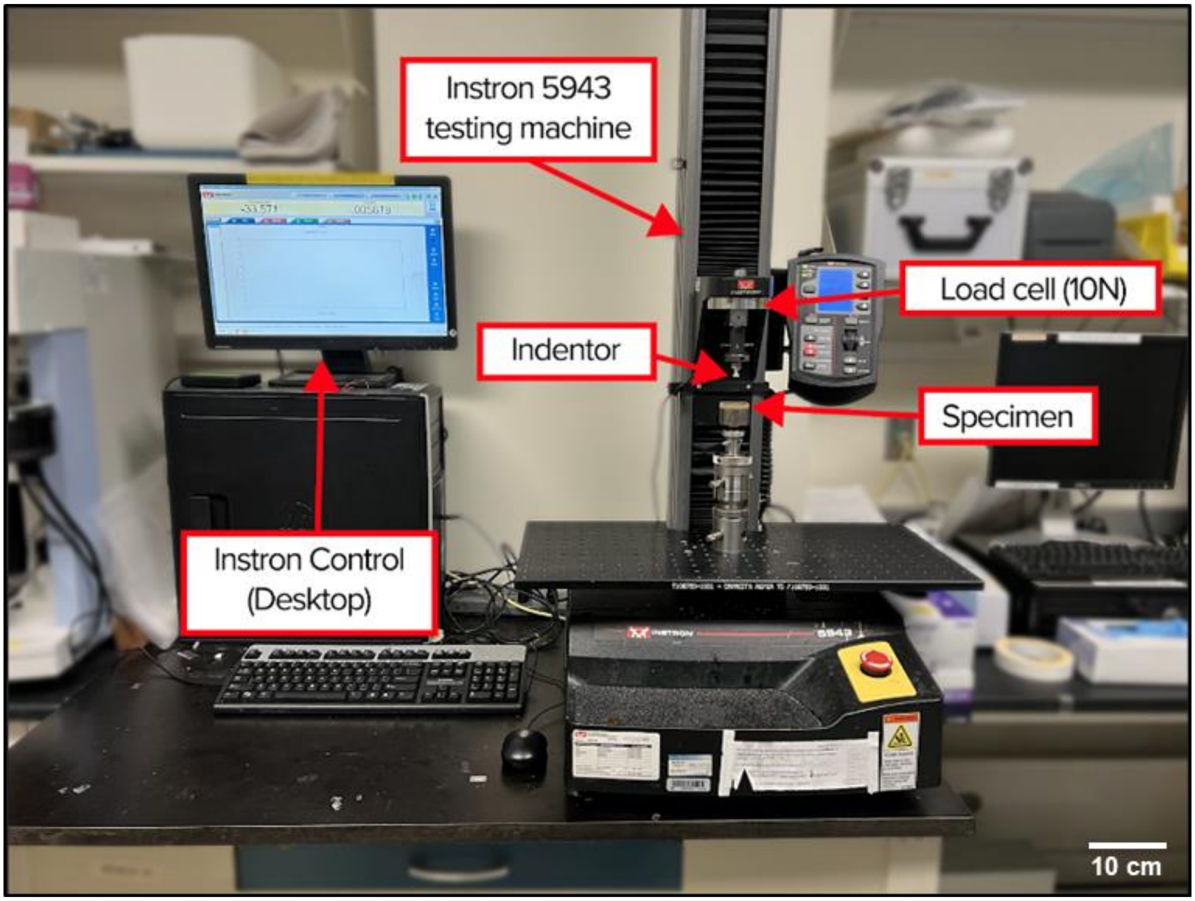

Supplementary Figure S8. Labeled photograph of mechanical compression testing setup.

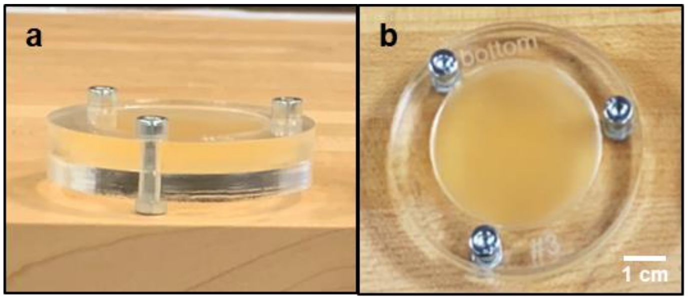

Supplementary Figure S9. a. Side view and **b.** top view of specimens prepared using a custom acrylic mold for mechanical and acoustic characterization. Prepared specimen have diameter of 4 cm and thickness of 6 mm.

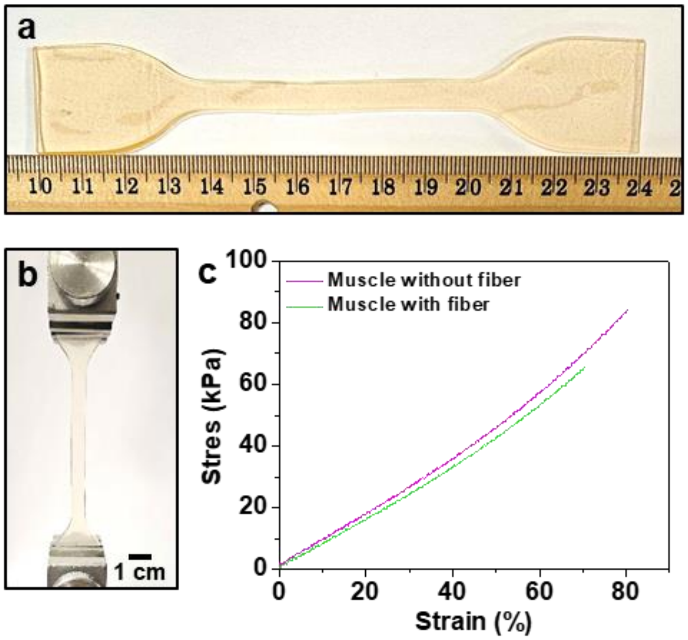

Supplementary Figure S10. a. Photograph of a dogbone-shaped specimen for tensile testing. **b.** Photograph of the specimen loaded for the tensile testing. **c.** Stress-strain curve of muscle TMM with and without the collagen-dextran fiber network obtained from tensile testing.

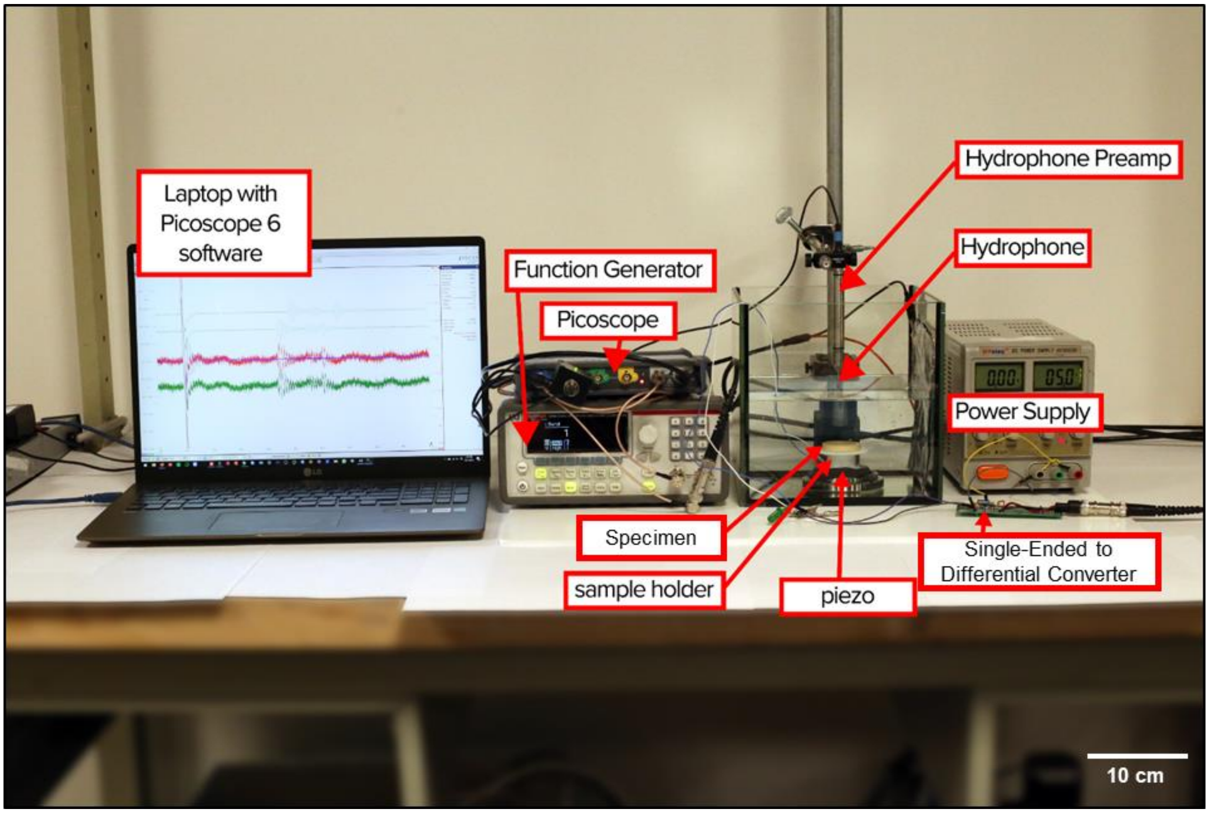

Supplementary Figure S11. Labeled photograph of acoustic testing setup.

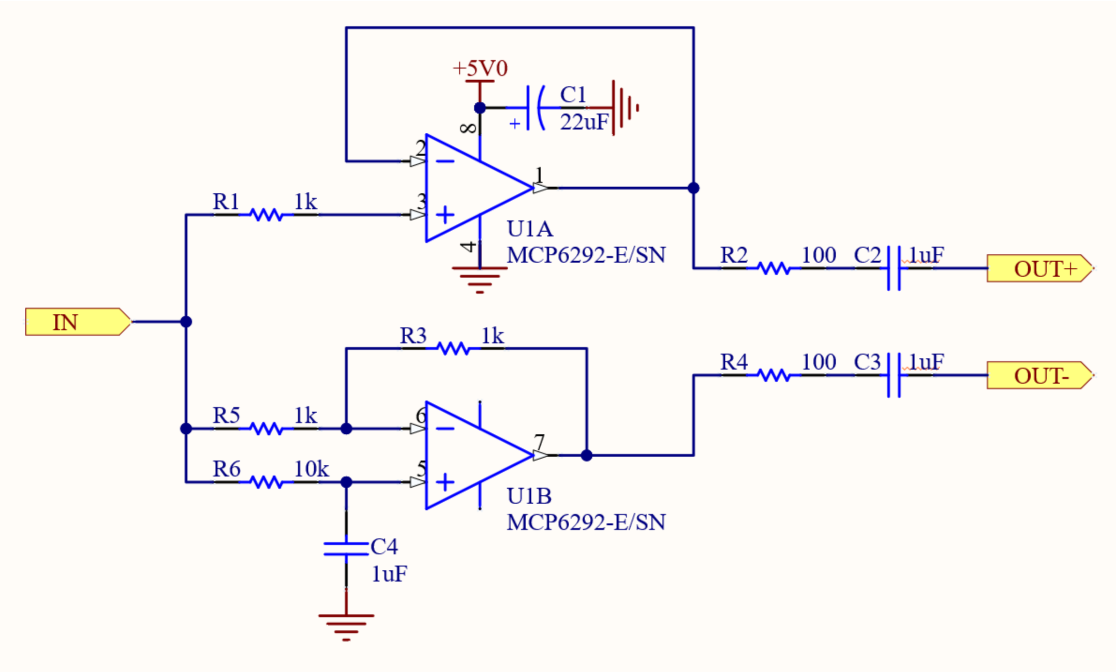

Supplementary Figure S12. Circuit diagram of single-ended differential converter used in pulse-echo measurement. This single-ended differential amplifier was used to reduce the electric field coupling.

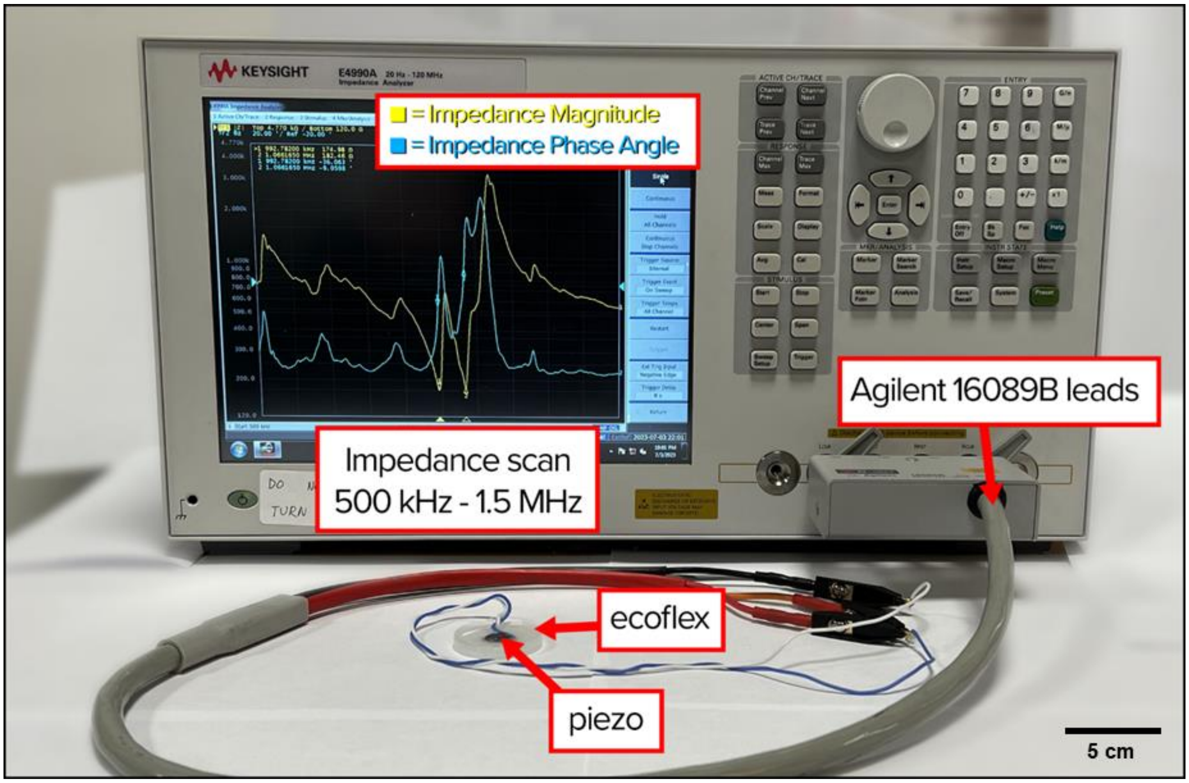

Supplementary Figure S13. Labeled photograph of piezoelectric transducer resonant frequency measurement setup using the impedance analyzer. The cyan numbers in the top left show the piezoelectric element's resonance frequency to be 1 MHz.

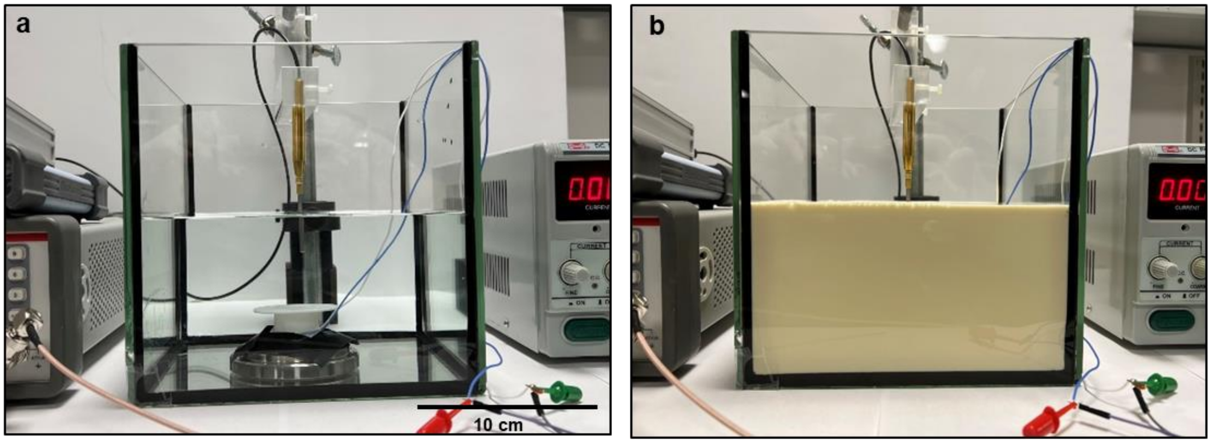

Supplementary Figure S14. Photograph of **a.** Pure water and **b.** Liquid TMM acoustic testing setup.

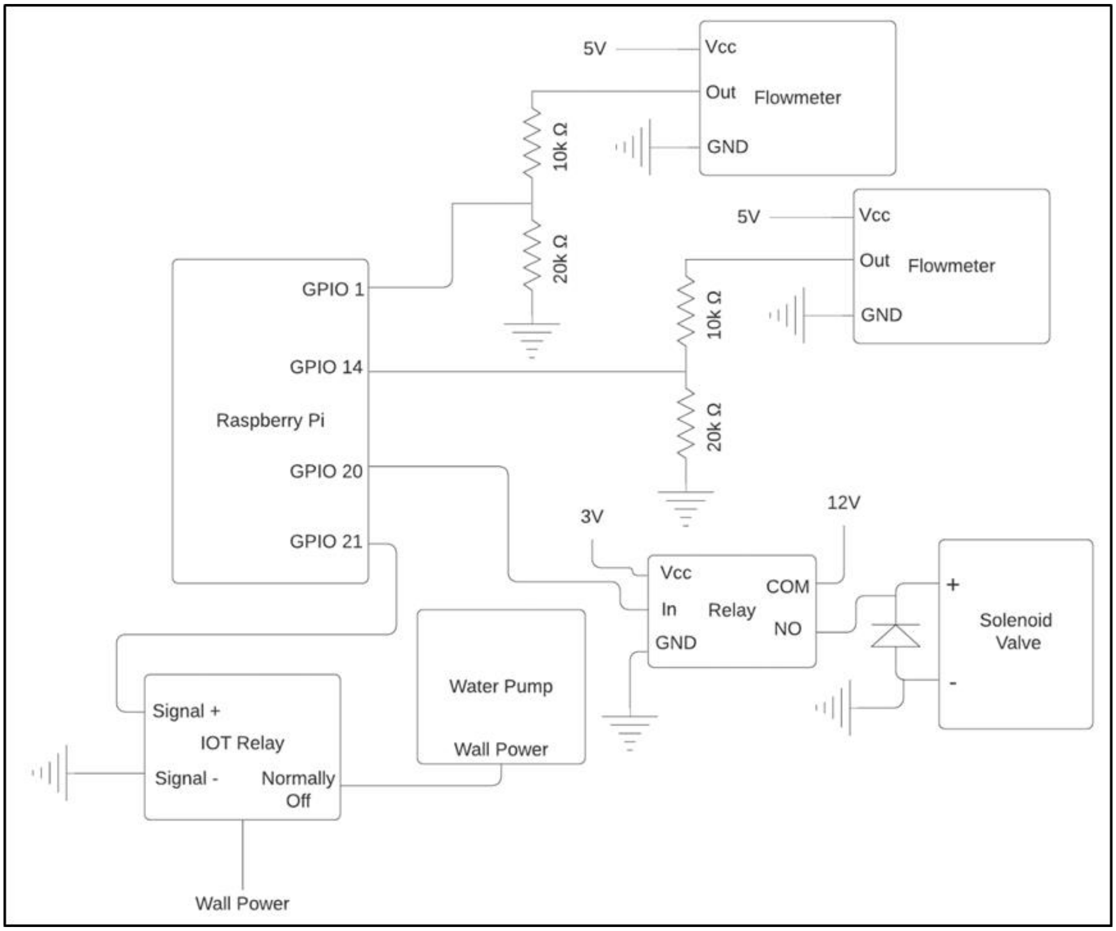

Supplementary Figure S15. Circuit diagram of electronics system that can programmatically control the bladder phantom volume and flow rate of liquid in the torso tank system.

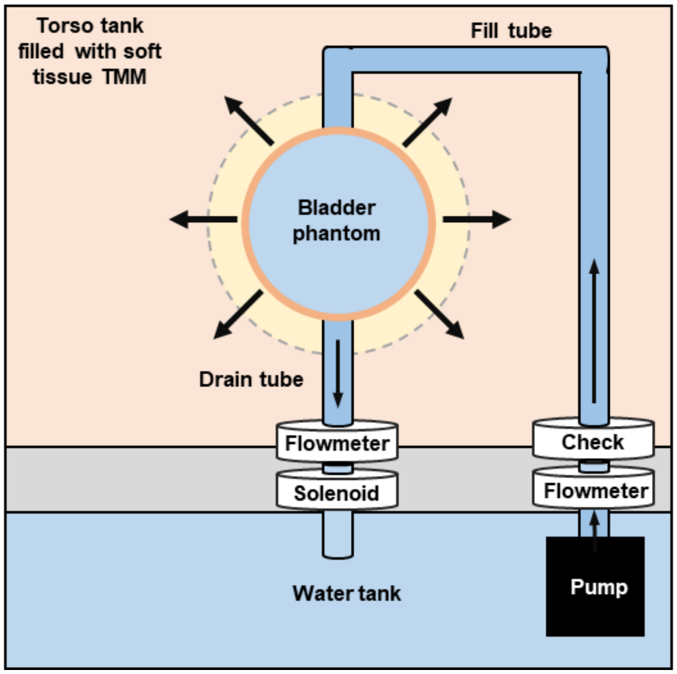

Supplementary Figure S16. Schematic description of electro-mechanical system in torso tank to fill (and expand) the bladder phantom. Two programmable flowmeters are connected to the inlet (Fill tube) and outlet (Drain) tube along with a programmable solenoid valve shown in Figure S14. These valves can digitally control the water flow to adjust the volume of the water entering and exiting the bladder phantom. The check valve ensures there is no backflow.

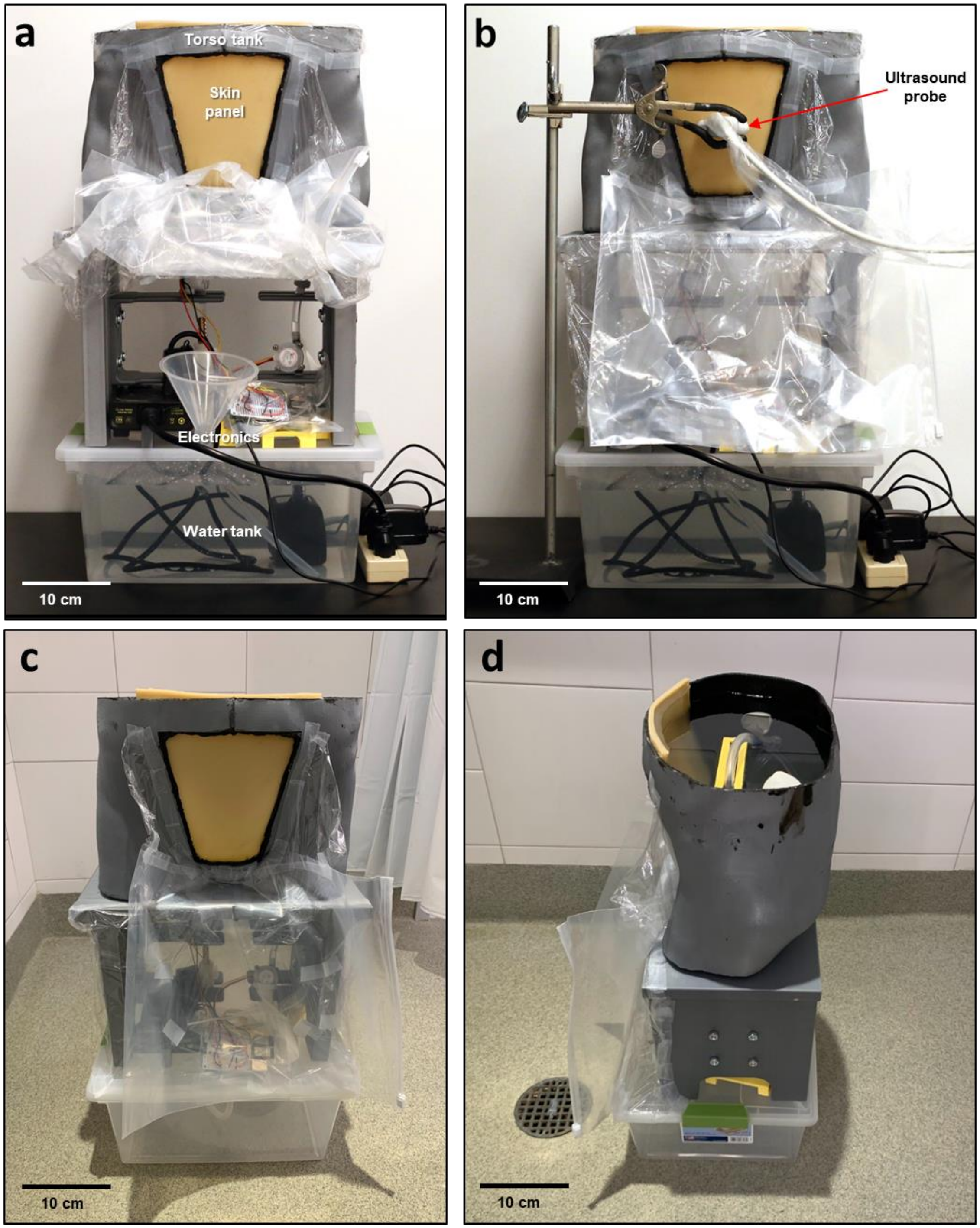

Supplementary Figure S17. a. Photograph of full torso tank system schematically shown in Figure 5a. **b.** Whole system with an ultrasound probe. Schematic description of the whole system. Photograph taken from **c.** tilted-front, and **d.** side view while doing waterproof test. Torso tank was filled with water and showed no leakage. Electronic components are covered by a plastic shield to prevent accidental water exposure.

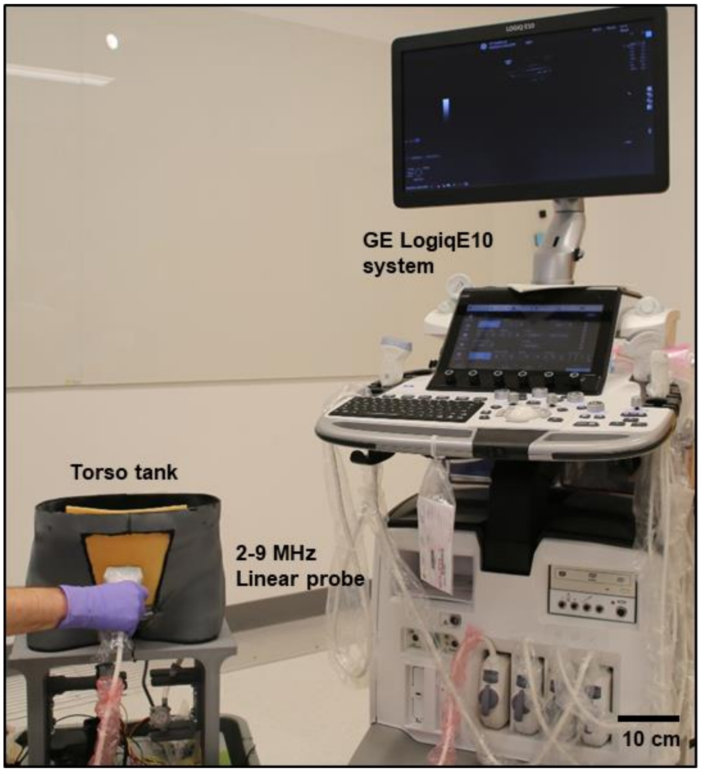

Supplementary Figure S18. Photograph of the torso tank system and a commercial ultrasound system for ultrasound imaging of the bladder phantom. A linear probe with a frequency of 2-9 MHz was used for the imaging.

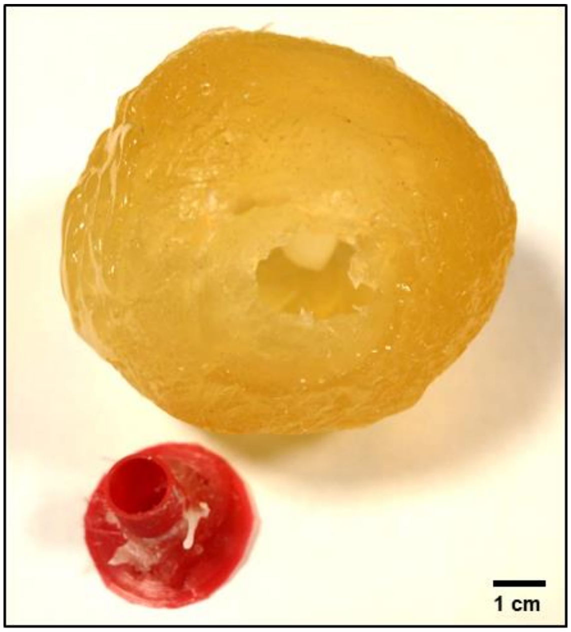

Supplementary Figure S19. Photograph of the broken bladder phantom during the bladder phantom expansion by the addition of water. The main failure point is the connector-phantom interface.

Supplementary Table S1. Comparison of various bladder phantoms from the literature.

	Materials	Young's modulus (kPa)	Density (g/cm3)	Speed of sound (m/s)	Attenuation (dB·cm^{-1}·MHz^{-1})	Expandable	Ability to fill and empty the bladder	Reference
Human bladder	-	70-430	1.04-1.18	1547-1616	0.23-1.09	Yes	Yes	[5-8]
Our work	Gelatin, psyllium, formalin, and water	81.6 ± 9.0	1.04 ± 0.02	1578.3 ± 4.8	0.5 ± 1.44	Yes	Yes	-
CIRS Inc.	Proprietary	4-90	0.94	1529.3 ± 2.9	0.54 ± 0.02	No	No	[9-11]
Ejofodomi et al.	Gelatin, polystyrene microsphere, and water	17.12 ± 2.72	1.05 ± 0.02	1591 ± 8.76	0.66 ± 0.08	No	No	[5]
Wognum et al.	Porcine bladder	16	-	-	-	Yes	No	[12-13]
Shellikeri et al.	Latex balloon	8250	1.165	-	-	Yes	No	[14-15]
Verstraeten et al.	Ecoflex 00-20	-	1.0661	973.6	0.43	Yes	No	[16-18]
Choi et al.	Ecoflex 00-30	100-125	1.0661	973.6	0.43	Yes	No	[17-20]

Supplementary References

[1] A. Fedorov, D. Clunie, E. Ulrich, C. Bauer, A. Wahle, B. Brown, M. Onken, J. Riesmeier, S. Pieper, R. Kikinis, J. Buatti, R. R. Beichel, *PeerJ* **2016**, 4, e2057.
[2] 3D Slicer image computing platform, https://www.slicer.org/, accessed.
[3] democratiz3D, https://www.embodi3d.com/democratiz3D/, accessed.
[4] Autodesk Meshmixer, https://meshmixer.com/, accessed.
[5] O. t. A. Ejofodomi, V. Zderic, J. M. Zara, *Med. Phys.* **2010**, 37, 1440.
[6] S. E. Dahms, H. J. Piechota, R. Dahiya, T. F. Lue, E. A. Tanagho, *Br. J. Urol.* **1998**, 82, 411.
[7] I. Foundation, Speed of sound, https://itis.swiss/virtual-population/tissue-properties/database/acoustic-properties/speed-of-sound/, accessed.
[8] P. Sam, A. Nassereddin, C. A. LaGrange, *Anatomy, Abdomen and Pelvis: Bladder Detrusor Muscle*, StatPearls Publishing, **2023**.
[9] M. Zerhouni, M. Rachedine *United States Patent 5,196,343*, **1993**.
[10] E. Çetin, H. O. Durmuş, B. Karaböce, N. Kavaklı, presented at 2019 IEEE International Symposium on Medical Measurements and Applications (MeMeA), 26-28 June 2019, **2019**.
[11] S. Nuclear, General Purpose Ultrasound Phantom, https://www.sunnuclear.com/products/zerdine-ultrasound-phantom, accessed.
[12] S. Wognum, S. E. Heethuis, T. Rosario, M. S. Hoogeman, A. Bel, *Med. Phys.* **2014**, 41, 071916.
[13] R. van Mastrigt, B. L. R. A. Coolsaet, W. A. van Duyl, *Medical and Biological Engineering and Computing* **1978**, 16, 471.
[14] S. Shellikeri, S. J. Back, L. Poznick, K. Darge, *Ultrasonud Med. Biol.* **2018**, 44, 1918.
[15] K. M. Lee, A. R. McNeese, L. M. Tseng, M. S. Wochner, P. S. Wilson, *Proceedings of Meetings on Acoustics* **2014**, 18.
[16] M. Verstraeten, G. Bou Kheir, L. Vande Perre, R. Raffoul, J. Chavez Cerda, A. Delchambre, T. Roumeguere, A. Vanhoestenberghe, A. Nonclercq, *Biomed Phys Eng Express* **2023**, 9.
[17] A. Cafarelli, P. Miloro, A. Verbeni, M. Carbone, A. Menciassi, *Journal of Ultrasound* **2016**, 19, 251.
[18] Y. Yamashita, Y. Hosono, K. Itsumi, presented at 2007 Sixteenth IEEE International Symposium on the Applications of Ferroelectrics, 27-31 May 2007, **2007**.
[19] E. Choi, F. Waldbillig, M. Jeong, D. Li, R. Goyal, P. Weber, A. Miernik, B. Grüne, S. Hein, R. Suarez-Ibarrola, M. C. Kriegmair, T. Qiu, *Ann. Biomed. Eng.* **2021**, 49, 2412.
[20] B. Herren, M. C. Saha, M. C. Altan, Y. Liu, *Composites Part B: Engineering* **2020**, 200, 108224.

Supplementary Movies

Movie S1. A video shows bladder phantom hanging in the air while filled with water.

Movie S2. A video shows bladder phantom filled with water and pouring the water in the bladder phantom.

Movie S3. A video shows bladder phantom filled with water and pouring the water in the bladder phantom.

Movie S4. A video shows operation of the torso tank with the bladder phantom under dynamic conditions.

Movie S5. An ultrasound video shows the bladder phantom with a programmable control of the bladder volume.
